# Pathways involved in pony body size development

**DOI:** 10.1186/s12864-020-07323-1

**Published:** 2021-01-18

**Authors:** Jun Fang, Dong Zhang, Jun Wei Cao, Li Zhang, Chun Xia Liu, Yan Ping Xing, Feng Wang, Hong Yang Xu, Shi Chao Wang, Yu Ling, Wei Wang, Yan Ru Zhang, Huan Min Zhou

**Affiliations:** College of Life Sciences, Inner Mongolia Agricultural University, No. 306 Zhaowuda Road, Hohhot, 010018 China

**Keywords:** Growth hormone receptor, Debao pony, RNA-seq, Short stature

## Abstract

**Background:**

The mechanism of body growth in mammals is poorly understood. Here, we investigated the regulatory networks involved in body growth through transcriptomic analysis of pituitary and epiphyseal tissues of smaller sized Debao ponies and Mongolian horses at the juvenile and adult stages.

**Results:**

We found that *growth hormone receptor* (*GHR*) was expressed at low levels in long bones, although g*rowth hormone* (*GH*) was highly expressed in Debao ponies compared with Mongolian horses. Moreover, significant downregulated of the *GHR* pathway components *m-RAS* and *ATF3* was found in juvenile ponies, which slowed the proliferation of bone osteocytes. However, *WNT2* and *PLCβ2* were obviously upregulated in juvenile Debao ponies, which led to premature mineralization of the bone extracellular matrix. Furthermore, we found that the *WNT/Ca*^*2+*^ pathway may be responsible for regulating body growth. *GHR* was demonstrated by q-PCR and Western blot analyses to be expressed at low levels in long bones of Debao ponies. Treatment with WNT antagonistI decreased the expression of *WNT* pathway components (*P* < 0.05) in vitro. Transduction of ATDC5 cells with a GHR-RNAi lentiviral vector decreased the expression of the *GHR* pathway components (*P* < 0.05). Additionally, the expression of the *IGF-1* gene in the liver was lower in Debao ponies than in Mongolian horses at the juvenile and adult stages. Detection of plasma hormone concentrations showed that Debao ponies expressed higher levels of IGF-1 as juveniles and higher levels of GH as adults than Mongolian horses, indicating that the hormone regulation in Debao ponies differs from that in Mongolian horses.

**Conclusion:**

Our work provides insights into the genetic regulation of short stature growth in mammals and can provide useful information for the development of therapeutic strategies for small size.

**Supplementary Information:**

The online version contains supplementary material available at 10.1186/s12864-020-07323-1.

## Background

Domestic animals’ body size is a crucial index for determination of horse breeds and has become a priority factor in animal breeding. It is closely related to their physiological function, production performance, disease resistance and adaptability to the external environment [1, 2]. Given the high value of this trait, in-depth investigations into its genetic aspects in domestic species have been conducted. To date, molecular elements related to body size have been investigated in pigs and cows as well as in humans [3–8]. The related studies have indicated that SNPs in the *GH1* gene [3] and haplotypes with a long sweep on X chromosome [4] are associated with body size in pigs. The growth pattern of body and organ in pigs with growth hormone receptor (*GHR*) knockout mutations are similar to those in human with Laron syndrome, which is a rare and autosomal recessive disorder caused by loss-of-function mutations in the *GHR* gene [5]. Cattle with the haplotype combination H3H3 (CC-GG-CC-AA-CC) which varied in the *STAT3* gene promoter regions is significantly enhanced body size than that with haplotype combination H1H1 (AA-AA-AA-AA-TT) and H2H3 (CC-GG-AC-GA-CC), respectively [6]. Whole-genome sequence analysis has shown that the genetic architecture of stature in cattle is similar to that in humans [7]. However, many more height-related genes have been identified in humans than in these other mammals [8].

Like all domestic animals, horses have evolved into many different populations with widely varying body sizes through natural and artificial selection. A few studies on the genetic aspects of body size in horses have been conducted. For example, a genome-wide association study based on SNPs identified two chromosomal loci near the *LCORL/NCAPG* gene and the *ZFAT* gene that have already been shown to influence body height in humans [9]. In addition, a whole-genome sequencing study on two miniature Shetland ponies and one standard-sized Shetland pony revealed four synergistic variants including in *ADAMTS17*, *OSTN*, *GHI* and *HMGA2* that limit wither height to 87 cm and seemingly reveal the main reason for the short stature of miniature ponies [10]. A complementary genome analysis of ponies and tall horses identified the genomic loci related to body height and metabolic traits and discovered that *HMGA2* c.83G > A (p.G28E) variants were significantly altered in Welsh ponies, suggesting that the highly related loci in the ponies were highly efficient in altering metabolic pathways [11]. However, body size is a complex quantitative trait controlled by multiple genes. Thus, the molecular pathways regulating body height in horses remain unclear.

Body size depends largely on long bone growth and endocrine hormone signaling. Bones themselves, as well as other endocrine organs can act synergistically to promote growth [12, 13]. In this study, we sequenced the transcriptomes of the pituitary gland and long bone tissues from Debao ponies (DPs) and Mongolian horses (MHs). The DP and MH are registered standard native horse breeds in China. The DP, which is less than 106 cm in height, originated in Debao County in the Guangxi Zhuang Autonomous Region of southwestern China and is well adapted to the local mountain environment. The MH, which is 122 cm to 142 cm in height, is one of the oldest horse breeds inhabiting the Mongolian Plateau; this breed is adaptable and exhibits strong disease resistance and hardiness on rough terrain [14, 15]. Previous reports have indicated that the DP has a genetic relationship with the MH [16] although both populations have undergone long-term natural and artificial selection over the course of their evolution that has resulted in significant differences in height. However, little is known about the molecular mechanism determining the body size of DPs. Hence, we systematically screened the key genes involved in the regulatory network of growth body size to reveal the correlation between the expression patterns of candidate genes and body size traits and to further elucidate the molecular pathways influencing body size.

## Results

### Identification of differentially expressed genes (DEGs) in DPs

Height analysis revealed obvious differences between DPs and MHs. DPs exhibited no significant differences in height between the juveniles (less than 3 years) and adults (more than 3 years) stages, while MHs did exhibit significant differences in height. DPs reached an the adult height at an early age, and the body length, chest circumference, canon bone circumference, neck length and head length increased similarly to the body height (Table 1). DPs and MHs exhibited significant differences in body height at the juvenile stage (*P* < 0.05) and at the adult stage (*P* < 0.005) (Additional file 1).

**Table 1:** Height Data from Debao Ponies and Mongolian **Horses**

NO.	Age (year)	Sex	Height (m)	Body length (m)	Bust (m)	Canon bone circumference(m)	Neck circumference (m)	Head circumference (m)
Pony1	1	female	1	0.95	1.1	0.13	0.49	0.4
Pony2	1	female	0.93	0.93	0.94	0.12	0.35	0.37
Pony3	1	female	1	0.93	1.02	0.14	0.4	0.37
Pony4	4	female	0.94	0.82	0.97	0.12	0.37	0.35
Pony5	5	female	0.88	0.85	0.94	0.13	0.4	0.36
Pony6	4	female	0.97	0.85	0.99	0.13	0.44	0.4
Horse1	1	female	1.27	1.29	1.4	0.06	0.65	0.52
Horse2	1	female	1.25	1.32	1.46	0.07	0.65	0.55
Horse3	1	female	1.24	1.25	1.39	0.07	0.61	0.52
Horse4	4	female	1.35	1.28	1.69	0.07	0.66	0.53
Horse5	4	female	1.37	1.43	1.6	0.07	0.71	0.54
Horse6	4	female	1.43	1.48	1.74	0.06	0.73	0.53

**Fig. 1:**
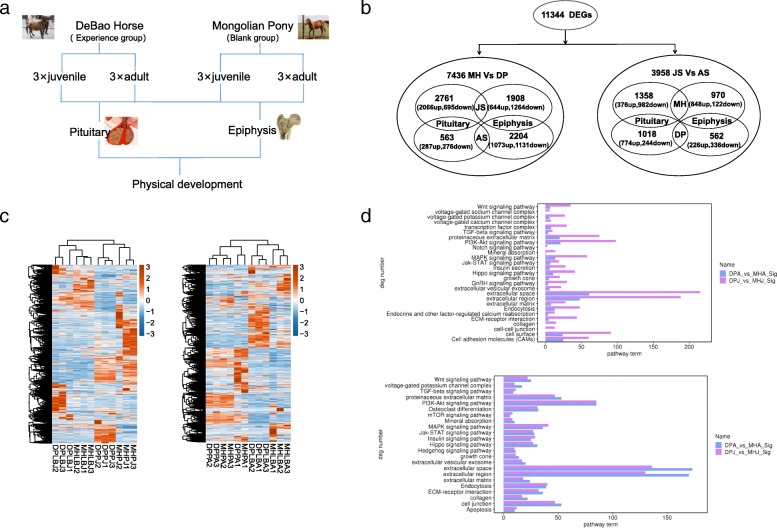
Summary of high-throughput sequencing data for the Debao pony (DP) and Mongolian horse (MH). **a** Schematic of the DP and MH groups. **b** Numbers of genes up/downregulated in the two breeds of horses at the two developmental stages (JS vs AS, juvenile stage vs adult stage). **c** Cluster analysis of the different developmental stages of DPs and MHs. The overall hierarchical clustering map based on FPKM values was generated with the log10 (FPKM+ 1) values. Red indicates strongly expressed genes, and blue indicates weakly expressed genes. The colors from red to blue indicated log10 (FPKM + 1) values from large to small. **d** GO and KEGG enrichment results for the DEGs in the MHPA vs DPPA and MHLBJ vs DPLBJ comparisons. MHPJ, juvenile Mongolian horse pituitary; MHPA, adult Mongolian horse pituitary; MHLBJ, juvenile Mongolian horse long bone; MHLBA, adult Debao pony long bone; DPPJ, juvenile Debao pony pituitary; DPPA, adult Debao pony pituitary; DPLBJ, juvenile Debao pony long bone; DPLBA, adult Debao pony long bone

We constructed and sequenced an RNA-seq library from 24 samples of pituitary and epiphyseal tissues taken from three DPs and three MHs at the juvenile and adult stages (Fig. 1a), as these tissue types are related to body size development [12, 13]. A total of 11,344 DEGs were obtained by RNA-seq, of which 7436 were differentially expressed between the two breeds at the same stage (juvenile or adult). Specifically, 2761 and 563 DEGs in the pituitary and 1908 and 2204 DEGs in the long bone epiphysis were identified between MHs and DPs at the juvenile and adult stages, respectively. In addition, 3958 genes were differentially expressed between the different developmental stages in the same breed; 1358 and 970 DEGs were identified in the MH pituitary and long bone epiphysis, while 1018 and 562 DEGs were identified in the DP pituitary and long bone epiphysis, respectively (Fig. 1b). The sample correlation results showed that the pituitary samples were clusterd together independent of breed and developmental stage, while almost all long bone samples (except MH adult sample 4) were clustered by breed (Additional file 2). Cluster analysis of the different developmental stages of DPs and MHs showed significant differences between pituitary and epiphyseal tissues in the two breeds at the two developmental stages (Fig. 1c).

Significant differences were found in matrix metalloproteinases (*MMPs*) and *collagen* in long bone tissues between adult and juvenile horses. Greater enrichment of relevant pathways was observed at the juvenile stage than at the adult stage. However, the *MAPK* signaling pathway was less enriched at the juvenile stage than at the adult stage. In addition, significant differences were found in the *WNT* signaling pathway, the *PI3K-Akt* signaling pathway, cell junctions and cell surfaces in the pituitary glands between adult and juvenile horses (Fig. 1d). This finding suggests that in DPs, the *MAPK* signaling pathway may participate in limiting long bone growth at the juvenile stage.

**Fig. 2:**
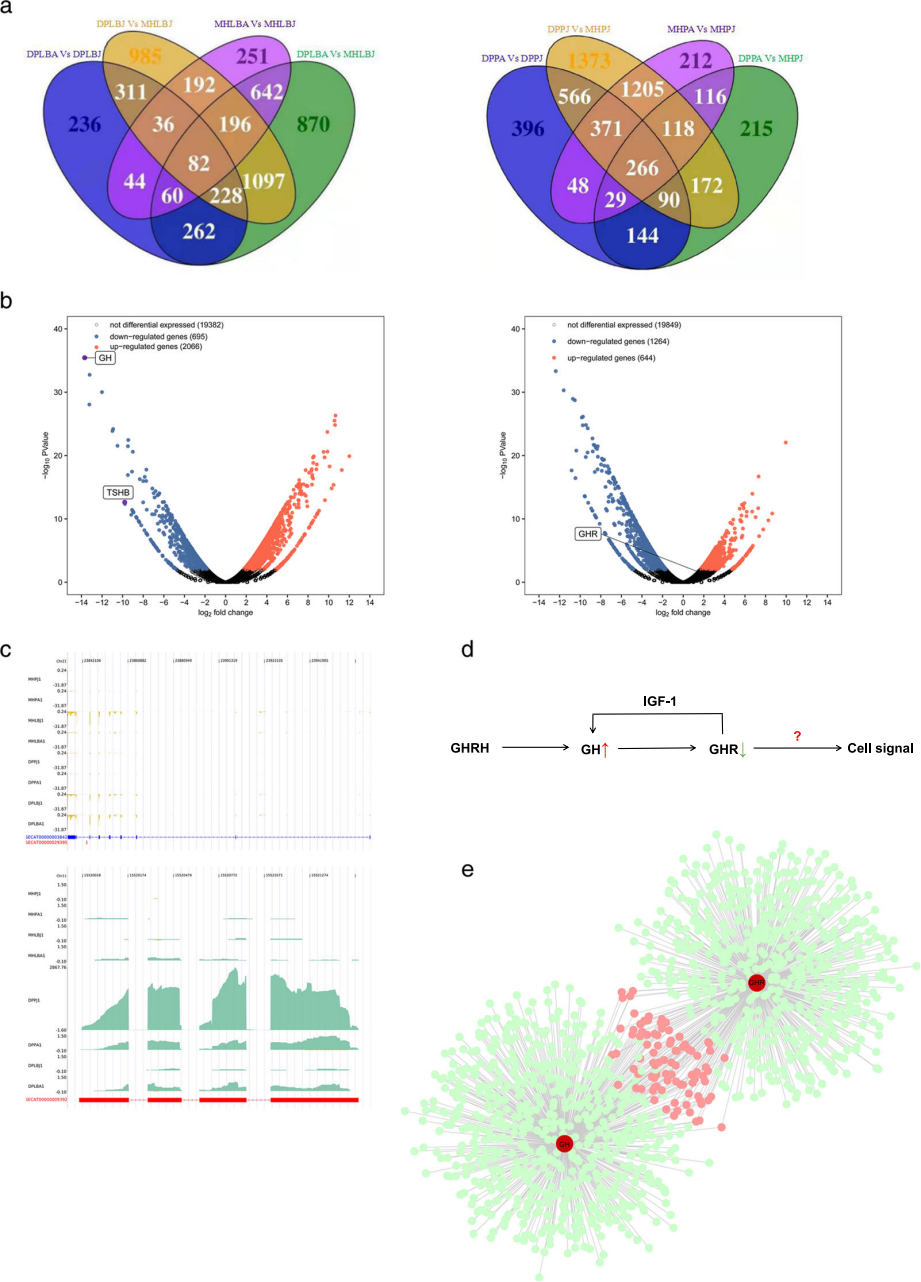
Expression levels of *GH* and *GHR* genes in pituitary and epiphyseal tissues from Debao ponies and Mongolian horses, as determined by RNA-seq. **a** Venn diagram showing the numbers of the DEGs in the comparisons between pituitary and epiphyseal tissues between the two breeds. **b** Volcano plots highlighting the DEGs in blue (*P* < 0.05) and red (q < 0.05) for the DPPJ vs MHPJ and DPLBJ vs MHLBJ comparisons DPPJ, juvenile Debao pony pituitary; MHPJ, juvenile Mongolian horse pituitary; DPLBJ, juvenile Debao pony long bone; MHLBJ, juvenile Mongolian horse long bone. **c** Integrative Genomics Viewer visualization of GH and GHR gene RNA-seq data from the DP and MH pituitary and long bone tissue samples. Red indicates the reference gene tracks (ENSECAG00000002986 GHR;ENSECAG00000009392 GH); yellow and blue indicate the GHR and GH gene RNA-seq data tracks, respectively; the Y-axis shows the different samples; the X-axis shows a genome coordinate ruler that indicates the size of the region considered. **d** Interactions of GH and GHR and their effects on downstream cell signaling pathways. **e** The coexpression network linking GH and GHR revealed several key genes, such as *ATF3*, *EGR3*, *SOX2*, *SOX5*, and *RUNX2*

As shown in the Venn diagram, 82 DEGs in the long bone epiphysis overlapped between the two breeds of horses at different stages, while 266 DEGs in pituitary tissue overlapped (Fig. 2a). The volcano plot shows that *GH* and *TSHB* were significantly upregulated in the pituitary tissues of juvenile DPs and MHs, while *GHR* was significantly downregulated in the long bones of juvenile DPs and MHs. *GHR* expression was significantly lower in DPs than in MHs. In addition, the expression of genes related to the epiphyseal cell matrix, such as *ALPL*, *COL* and *MMP* was significantly higher in MHs than in DPs, while the expression of *GH*, *TSH* and *IGF-1* in the pituitary was significantly higher in juvenile DPs than in juvenile MHs (Fig. 2b).

### DPs exhibit excessive *GH* expression and low *GHR* expression

*GH* expression and *GHR* expression were differed significantly in the tissues of the two breeds (Fig. 2c). *GH* expression in the juvenile DP pituitary (DPPJ) was 13-fold higher than that in the juvenile MH pituitary (MHPJ) (Additional file 3). Although *GHR* expression in the juvenile DP long bone (DPLBJ) was 1.4-fold lower than that in the juvenile MH long bone (MHLBJ) (Additional file 4). Analysis of protein levels showed that GH expression in the pituitary tissue was higher in DPs than that in MHs at both stages, although this difference was not found in long bone tissues (Additional file 5). Thus, we found that DPs have high expression of *GH* and low expression of *GHR*. Low expression of GHR may lead to GH insensitivity. Previous studies have shown that idiopathic dwarfism is associated with extremely low expression of GHR [17, 18]. Thus, we hypothesized that the low expression of GHR may be related to the small size of DPs.

GH is synthesized and secreted mainly by GH cells in the anterior pituitary and is very important for the growth and development of bone and the maintenance of bone mass. When GH and GHR are expressed simultaneously, they act on target cells through a corresponding signaling pathways (Fig. 2d). Through analysis of the coexpression network linking *GH* and *GHR*, we found that many genes related to extracellular matrix (ECM) development were associated with these cellular signaling pathways (Fig. 2e).

**Fig. 3:**
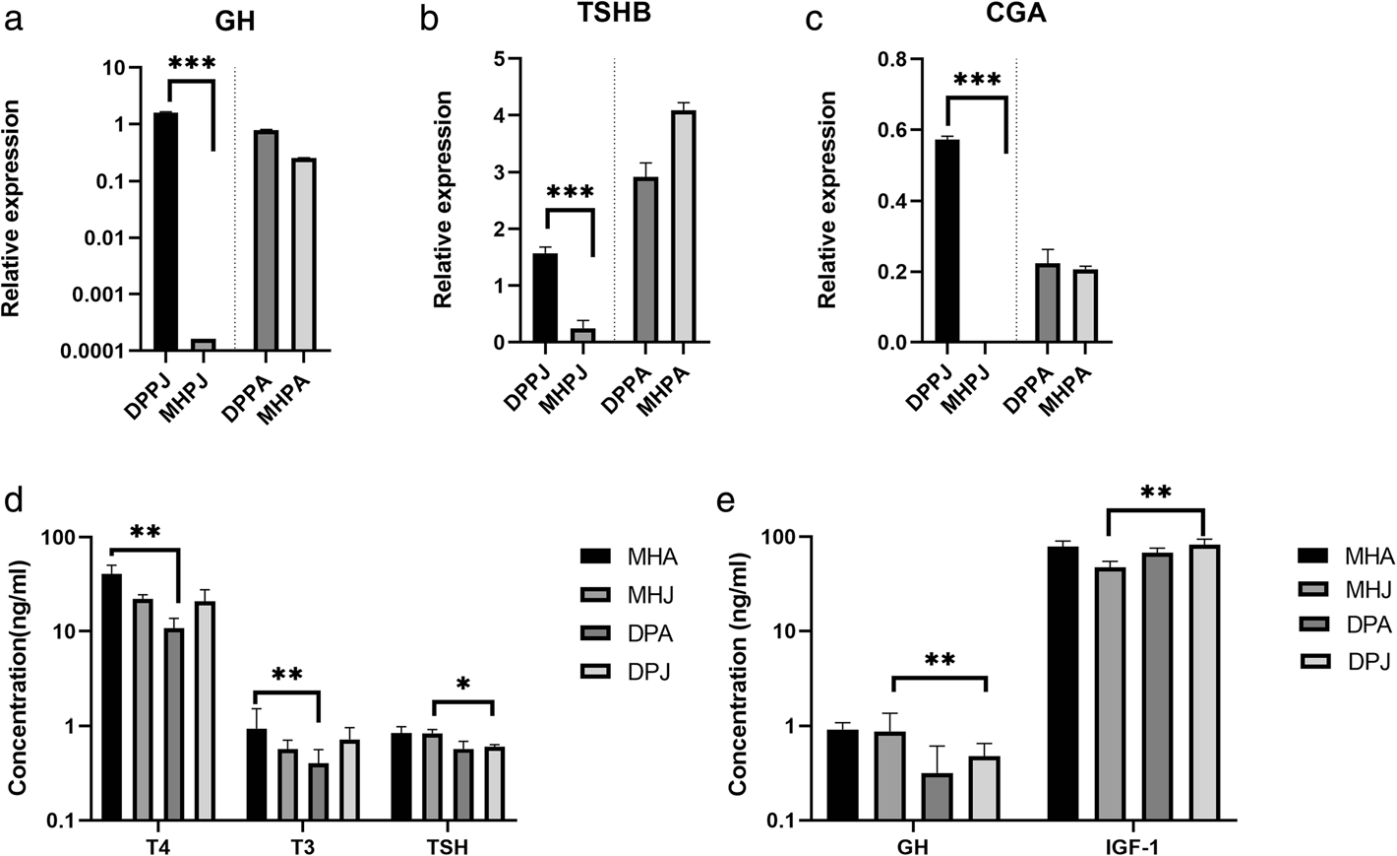
Expression levels of key genes in pituitary tissues from Debao ponies and Mongolian horses. **a** Expression levels of GH genes in pituitary tissues from Debao ponies and Mongolian horses. **b, c** Expression levels of *TSHB* and *CGA* in pituitary tissues from Debao ponies and Mongolian horses. **d** Determination of plasma T3, T4, and TSH concentrations in Mongolian horses and Debao ponies during the juvenile and adult stages. **e** Determination of plasma GH and IGF-1 concentrations of in Mongolian horses and Debao ponies during the juvenile and adult stages. (*P < 0.01, **P<0.05, ***P<0.001)

GH stimulates the production of IGF-1, and IGF-1 acts as a surrogate marker for GH. The results of this study revealed that the expression of *IGF-1* was higher in the liver in MHs than in DPs at both the adult and juvenile stages (Additional file 6). In addition, GH expression was extremely high in the DP pituitary of DPs compared with the pituitary of MHs at the juvenile stage (*P* < 0.001) (Fig. 3a). Transcript levels alone are not sufficient to predict protein levels in many situations. In additon, other hormones have been shown to be different in the two horse breeds. For example, common glycoprotein alpha (CGA) and TSHB are positively and negatively regulated by triiodothyronine (T3), respectively. We found no significant differences in CGA or TSHB expression between adult DPs and MHs; however, the opposite result was observed in juvenile horses (Fig. 3b, c).

To determine the effects of additional relevant hormones on the body height of DPs, the concentrations of T3, T4, IGF-1, GH and TSH in plasma were determined by radioimmunoassay. Plasma hormone concentrations differed significantly among the groups. The T3 and T4 concentrations were higher in adult MHs than in DPs (*P* < 0.001), but did not differ at the juvenile stage in these two breeds; the T3 and T4 concentrations in adult MHs were significantly higher than those in juvenile MHs (*P* < 0.001), while those in DPs displayed the opposite trend; and the TSH content in juvenile DPs was lower than that in MHs (*P* < 0.01) (Fig. 3d). Moreover, the concentrations of GH and IGF-1 differed between the two horse breeds at the juvenile stage. In DPs, the IGF-1 and the GH concentrations were higher in juveniles than in adults (*P* < 0.01 and *P* < 0.001, respectively). However, in MHs, the plasma concentration of GH in juveniles was similar to that in adults, while the IGF-1 concentration was higher in adults (*P* < 0.001) (Fig. 3e). The different changes in hormone concentrations between these groups indicated that these genes may play an important role in the hypothalamus in regulating body size.

### In vitro validation of the key genes in signaling pathways related to short stature

**Fig. 4:**
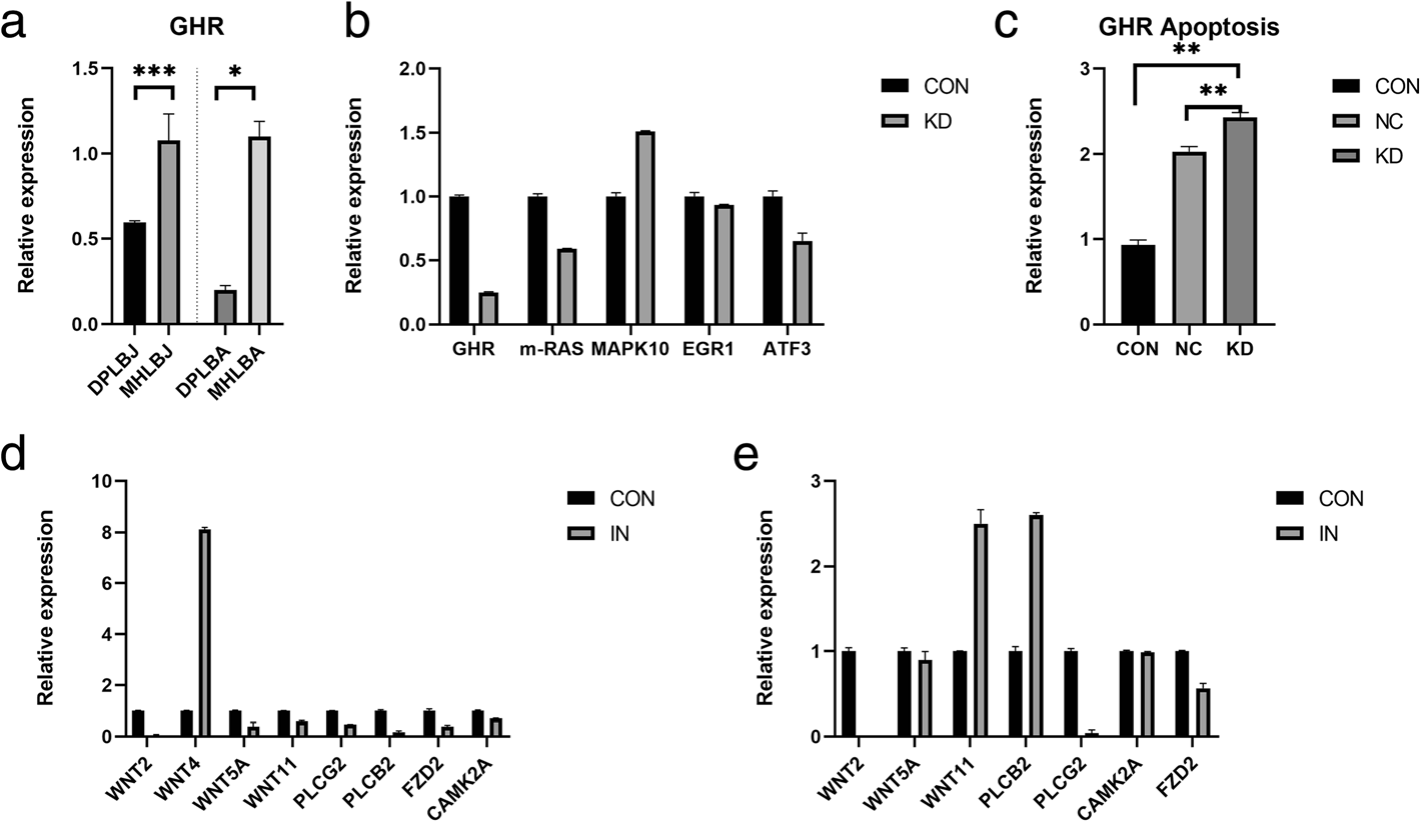
Expression levels of key genes in the long bone epiphyseal tissues from Debao ponies and Mongolian horses. **a** Expression levels of *GHR* in long bone tissues from Debao ponies and Mongolian horses. **b** Expression of the *GHR* signaling pathway in a lentivirus-infected cell line. **c** Detection of apoptotic cells by FACS. **d** Gene expression in the *WNT5A* signaling pathway in osteoblasts. **e** Gene expression in the *WNT5A* signaling pathway in ATDC5 cells. (*P < 0.01, **P < 0.05, ***P < 0.001)

According to the above experimental results, the *GHR* and *WNT* signaling pathways are relevant to the regulation of body growth. To obtain a reliable dataset for RNA-seq analysis, we conducted a series of in vitro experiments. Knockout or silencing of key genes to observe phenotypic changes is the primary strategy for verification experiments. *GHR* expression in long bone tissue was much lower in juvenile DPs than in MHs (*P* < 0.001) (Fig. 4a). Moreover, targeted GHR-RNAi constructs were selected for packaging according to the characteristics of the ATDC5 cell line from mice. When ATDC5 cells reached 80% confluence, the optimal multiplicity of infection (MOI) was approximately 10 (Additional file 7). Lentiviral knockdown experiments showed that *GHR* expression in ATDC5 cells was significantly decreased after transfection (Additional file 8). The expression of the *GHR*, *m-RAS* and *ATF3* genes, which are related to the *GHR* pathway, in transfected cells was lower than that in untransfected cells (*P* < 0.05) (Fig. 4b). Furthermore, the apoptosis rate of the control cells was lower than that of the untransfected cells, and the apoptosis rate of the blank cells was lower than that of the *GHR*-knockdown cells. In contrast, the apoptotic rate of virus-transfected cells was relatively high (*P* < 0.05) (Fig. 4c, Additional file 9).

The role of the *WNT* signaling pathway was verified in two cell lines, one of which was a horse bone marrow mesenchymal stem cell (BMSC) line. The other cell line used for validation was ATDC5. First, the cell line was obtained and identified: Highly active horse BMSCs were obtained, and the expression of the transcription factor *Nanog* and the surface markers *CD44*, *CD90*, and *CD105* in the cell line was determined. After induction and differentiation, the resulting osteoblasts were determined to be in good condition and became nodular (Additional file 10). Alizarin red stained the bone nodules formed by osteoblasts red, and Alcian blue stained the regions with accumulated proteoglycans and hyaluronic acid blue (Additional file 10). The expression of *COL* and *ALPL* in osteoblasts increased significantly with the increasing induction time (Additional files 11 and 12). Second, the expression of *WNT* pathway genes in osteoblasts induced for 14 days was detected by quantitative real-time PCR (q-PCR) (Additional file 13). The expression of *WNT5A* and *WNT2* gions osteoblasts decreased significantly (*P* < 0.05). However, *CAMK2A* expression did not change significantly, suggesting that the noncanonical *WNT* pathway might not be altered in the bone tissue of DPs (Fig. 4d). The expression of the *WNT* pathway genes in the ATDC5 cell line was similarly detected by q-PCR (Additional file 14), *WNT4* expression was increased, although *WNT11, WNT5A* and *WNT2* expression was decreased (*P* < 0.05); additionlly, *FZD2*, *PLCɡ2* and *PLCβ2* expression was decreased in this cell line (Fig. 4e). In summary, these in vitro cell validation assays showed that the *WNT5A* and *WNT2* genes acted through the *PLCβ2* pathways via *WNT* antagonists in ATDC5 cells and horse BMSCs.

**Fig. 5:**
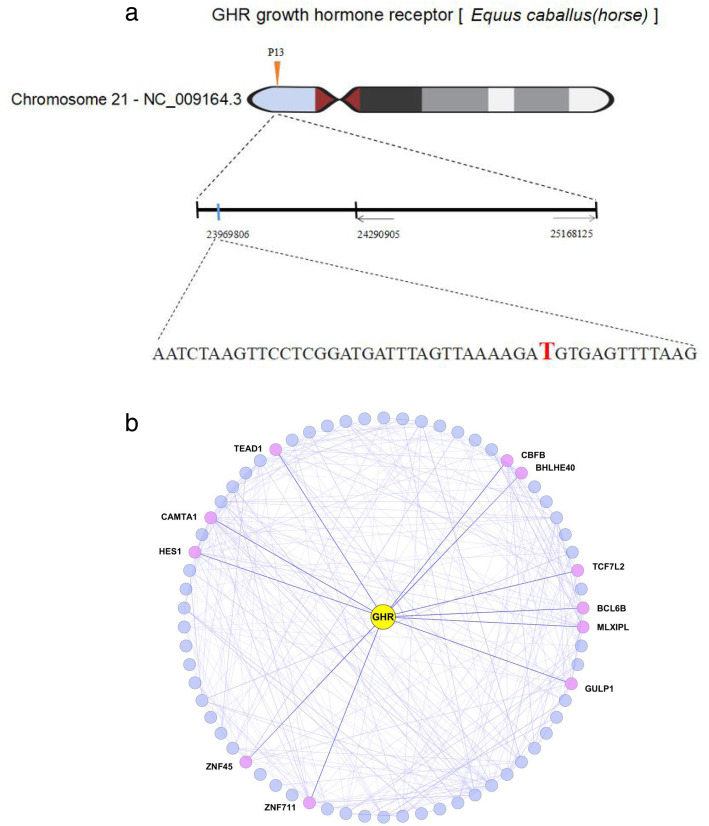
Transcription factor genes that coexpressed with *GHR* in Debao ponies and Mongolian horses. **a** Transcription factor genes coexpressed with *GHR* and coexpression relationships in the long bone tissues from the two breeds. **b** Location of the SNP in the *GHR* of the Debao pony

In addition, we investigated whether alterations in upstream transcription factors or in SNPs in the key gene *GHR*. The sequences of all transcription factor genes from *Equus caballus* (Assembly EquCab3.0) were obtained from an animal transcription factor database (http://www.bioguo.org/AnimalTFDB). In total, 71 transcription factor genes were coexpressed with *GHR,* and 11 of these genes might play important roles in regulating the expression levels of *GHR* (Fig. 5a). In our study, the 5000 bp nucleotide sequence upstream of the *GHR* transcription start site was investigated in DPs and MHs. One SNP, a non-synonymous mutation chr21 23,969,806 C-T of GHR (Fig. 5b) was found in DPs. Binding motifs analysis was performed, and the results revealed that SNPs led to differences in transcription factor binding motifs. This result implies that the SNP in *GHR* promoters might alter the binding motifs and lead to the different gene expression levels in the two breeds.

### Activation of bone signaling cascades is significantly altered in DPs

**Fig. 6:**
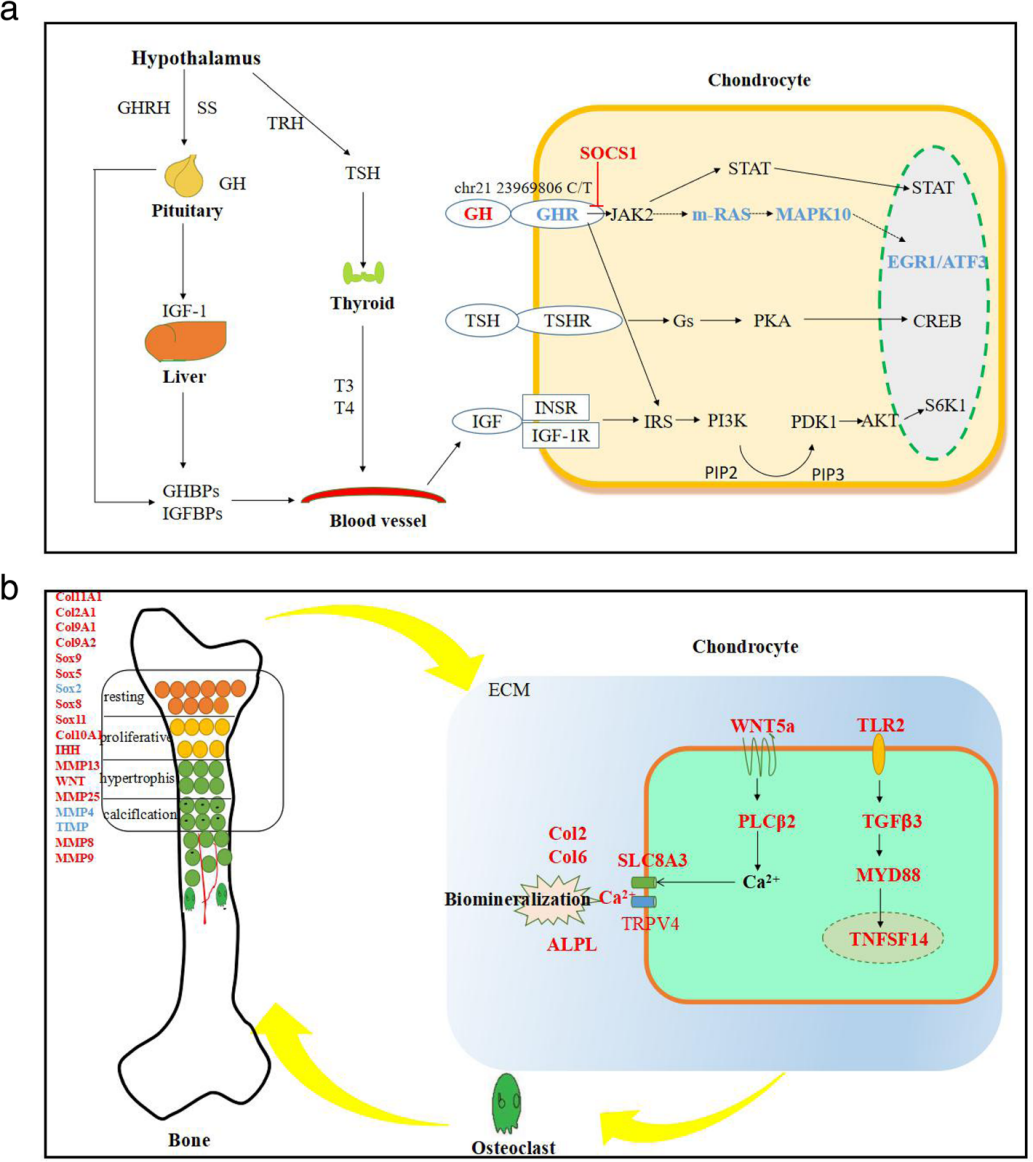
Signaling pathways regulating short stature in the Debao pony. **a** The *GHR* signaling pathway regulates short stature in the Debao pony. **b** The *WNT5A* and *TLR2* signaling pathways regulate short stature in the Debao pony. Genes with upregulated expression compared with that in the juvenile Mongolian horse are shown in red, while genes with downregulated expression are shown in blue

On the basis of the transcriptome sequencing results between DP and MH pituitary and epiphyseal tissues, candidate genes were screened, and q-PCR and Western blot analysis were the used to verify the accuracy of the transcriptome data (Additional files 3, 4, 5 and 6). The transcriptome data were considered from a biogenetics perspective to determine the possible regulatory pathways controlling short stature in DPs. The above results indicated that in juvenile DPs, the pituitary gland secretes high levels of GH, while the epiphyses exhibit a lack of *GHR*. *GHR*, *m-RAS* and *ATF3,* which are involved in the *GHR* pathway, were found to be significantly down regulated, by 75.2, 41 and 34.9%, respectively, in ATDC5 cells with GHR-RNAi lentivirus-mediated knockdown. Thus, bone growth in DPs may be inhibited via downregulation of the *GHR* pathway (Fig. 6a). However, *WNT2,WNT5A,PLCɡ2* and *FZD2* were noticeably downregulated, by 96.5, 61, 53.3 and 61%, respectively, in transfected ATDC5 cells. Such changes may increase the concentration of Ca^2+^ through *WNT* pathway activation and Ca^2+^ export from cells via solute carrier family 8 member A3 (*SLC8A3*) and transient receptor potential cation channel subfamily V member 4 (*TRPV4*) channels promote mineralization and early epiphyseal closure in coordination with changes in the expression of *ALPL* and epiphyseal factors, and stimulate osteoclastogenesis to enhance bone resorption via the *TLR* pathway (Fig. 6b).

## Discussion

Since 2007, when a draft genome of a thoroughbred mare was obtained, research on horses has entered a new era [19]. Gene expression levels in tissues from 8 breeds of horses were studied, and 75,116 transcripts were found, among which 20,302 protein-coding gene loci were accurately identified [20, 21]. A high-throughput study on body size correlations in horses confirmed that many genes are closely related to body height [9–11]. In addition, a genome-wide scan of the X chromosome of the DP using an Equine SNP70 BeadChip revealed that five regions on the X chromosome are under strong selection. The candidate regions include *SMS*, *DKC1*, etc., which are involved in bone development, GH secretion and fat deposition; these genes may also be related to body height [22]. Furthermore, an Equine 70 K SNP genotyping array was used for genome-wide detection of copy number variations among domestic DPs, MHs and Yili horses, and 60, 42 and 91 genes were found to overlap with the breed-specific, respectively. Thus, these genes may be relevant to breed-specific traits [16]. A study using an Equine SNP 65 BeadChip also revealed that a new candidate gene, T-box transcription factor 3 (*TBX3*), exhibited the greatest differentiation and most significant association with body size among the examined genes and is thus likely to be the dominating factor controlling the small stature of the DP. *TBX3* was elected independently in the DP, suggesting that there were multiple origins of small stature in horses [23]. However, these studies did not clearly reveal the molecular mechanism underlying short stature in the DP.

In this study, we selected MHs and DPs with different body heights for RNA-seq analysis and found that the expression levels of multiple genes were related to the heights of the two breeds. *GHR* expression in long bone tissue was 1.4-fold lower in DPs than in MHs, while *GH* expression was 13-fold higher, similar to the changes observed for dwarfism syndrome caused by familial dwarfism, idiopathic dwarfism and *GHR* mutation [17, 18] (Additional files 3 and 4). The routine treatment for these diseases is injection of recombinant GH, but this often causes transient hyperglycemia, peripheral edema, fluid retention and other side effects. Therefore, research on short- stature animals with normal physiological activities can reveal the physiological mechanism underlying the small size phenomenon and aid in the development of treatments for dwarfism and other diseases.

In many patients, the short stature is caused by an imbalance in changes in the body’s growth axis. The GHRH-GH-IGF axis, which is regulated by neuroendocrine factors, affects the growth and development of mammals. We thus collected the blood from these two breeds and measured hormone concentrations. We found that DPs exhibited unique physiological characteristics during the development of short stature, including high plasma IGF concentrations, low plasma GH concentrations, and high tissue GH expression levels. The results regarding plasma hormone levels were similar to those obtained for pygmies in Africa [24], but significantly different from those obtained for *GHR-*knockout animal models [5, 25]. This discrepancy may be due to the differences between knockout mice and natural dwarfs. Notably, thyroid hormone is an important regulator of bone growth [26]. Our transcriptome data showed a significant difference in TSH levels but no difference in TSHR levels between the two horses at the juvenile stage. Specifically, the plasma TSH levels in juvenile DPs were lower than those in juvenile MHs, while the plasma T3 and T4 concentrations in adult DPs were higher than those in adult MHs. The expression of CGA in tissues was consistent with that of TSH [27]. CGA regulates synthesis and secretion by affecting T3 and indirectly mediates the role of TSH in DPs. These results indicated that a change in the TRH-TSH-T3T4 growth axis might be contributing of short stature in DPs; however, the expression of *TSHR* in the pituitary tissue of juvenile DPs was not altered. Therefore, TSH may not be the main driver of short stature in DPs.

The hormones secreted by the pituitary gland and the development of long bones directly determine the body size of animals. We found that *GH* was highly expressed in pituitary and long bone tissues and that a lack of *GHR* expression in long bones may be the main cause of short stature in DPs. Although all *GHRs* function entirely through *GH* signaling, they still cannot meet the needs for bone growth in juvenile DPs, which may lead to the short stature of these horses. This pattern of *GHR,* characterized by low expression and deficiency, is similar to that observed in many dwarfism diseases [17, 18]. Most studies on body size have addressed the physiological characteristics of GHR deficiency, but few have explained the causes of GHR deficiency. We further studied the SNPs in *GHR* and found that in DPs, *GHR* harbors SNP loci that may lead to its altered transcription. The *GHR* SNP in DPs identified in this study was located in the promoter region, and it may be influenced by environmental factors or epigenetic factors, including methylation, in evolutionary genetics. Therefore, we speculate that compared with other horses of normal body size, DPs may exhibit not only polymorphisms in key genes in the growth axis but also alterations in the relevant important signaling pathways.

The transcriptome data obtained in this study showed a significant difference in the expression of Suppressor of Cytokine Signaling 1 (SOCS1) in bone tissue between juvenile DPs and MHs and that the expression of *SOCS1* was obviously increased in DPs. Moreover, *SOCS* family members, most prominently *SOCS1*, were upregulated in epiphyseal tissues of DPs. SOCS1 promotes ubiquitin-mediated degradation of JAK2 [28]. *SOCS2* is a key regulator of GHR sensitivity and is a GH-stimulated, STAT5b-regulated gene that acts in a negative feedback loop to downregulate *GHR* signaling [29].

Recent studies have made major strides in elucidating the mechanism of human JAK2 tyrosine kinase activation by GHR [30]. No significant differences in gene expression related to this pathway were identified in our study. However, we found that *GH* and *GHR* may reduce the height of DPs by downregulating the *MAPK* signaling cascade. The *MAPK* pathway is responsible mainly for the transcription of *ATF3*, and studies have shown that *ATF3* has a proapoptotic effect [31, 32]. In addition, the RNA-seq data revealed that the expression of *EGR1*, a zinc finger transcription factor-encoding gene located in the commonly deleted region (CDR) on chromosome 5q, was also decreased by the RNA-seq data. *EGR1* has been found to play a role in promoting apoptosis or inhibiting growth in many cancer studies [33, 34]. Perhaps these two transcription factors inhibit the proliferation and transformation of chondrocytes in each region of the cartilage by reducing apoptosis, leading to slow chondrocyte growth and abnormal development.

Bone tissue development involves numerous signaling molecules and signal transduction pathways. These include mainly the bone morphogenetic protein (BMP), TGFβ1 and WNT protein families. We found that *WNT5A* and *FZD2* expression in DPs was significantly upregulated compared with that in MHs (Fig. 4d). These genes belong to the noncanonical *WNT/Ca*^*2+*^ pathway, which is the main cell signaling pathway leading to Ca^2+^ deposition [35]. Thus, the levels of both intracellular and extracellular Ca^2+^ in DPs may be increased through the noncanonical *WNT* signaling pathway, leading to epiphyseal closure and cessation of growth. The expression of *SLC8A3*, *TRPV4, TRPV5* and *ALP* in the long bone tissue of juvenile DPs was also obviously increased (Fig. 6b). *SLC8A3* and *TRPV4/5* are the key genes encoding Ca^2+^ transport channels on the cell membrane [36, 37]. *ALP* is the decisive factor leading to bone mineralization [38, 39]. High expression of these genes in long bone tissue cells may increase Ca^2+^ transport, thus accelerating the mineralization of the osteoblast ECM.

In addition, significant changes were identified in *IHH, MMP23/25/8/9/11, TIMP4, COL10A1, SOX9/6/5/8/11, COL2A1, COL9A1/2, COL11A1, SOX5, MMP11* and *TIMP4*. Except for *SOX5, MMP11* and *TIMP4*, these genes were upregulated. The changes in these genes were consistent with changes in genes involved in cartilage development [40, 41]. These results showed that the cells in the four regions of the epiphyseal plate changed rapidly under stimulation by the corresponding factors (Fig. 6b).

Studies have shown that the *TLR2* and *MyD88* pathways play an important role in bone loss caused by infection [42, 43]. Our results showed that the genes in inflammation-related signaling pathways were upregulated. *TNFSF14* is also a member of the tumor necrosis factor receptor superfamily [44]. The protein encoded by this gene can promote transcription-related activation of proteins in osteoclasts, lead to the proliferation, growth, maturation and activation of osteoclasts, inhibit the proliferation and differentiation of osteoblasts, and promote the apoptosis of osteoblasts [45].

Molecular experiments showed that the collected horse bone marrow cells expressed the stem cell transcription factor *Nanog* and surface markers *CD44*, *CD90* and *CD105*. According to previous literature, these markers are characteristic of BMSCs [46, 47]. q-PCR assays confirmed that *COL2A1*, *COL1A2*, *COL6A1* and *ALPL* (Additional file 11) were expressed in induced osteoblasts. As the induction time increased, the expression of most genes increased gradually, while *COL1A2* expression peaked its maximum on the 21st day, consistent with findings of Yoo et al. regarding *COL* expression [48]. The expression of *ALPL* is used to evaluate the activity of osteoblasts, and high *ALPL* expression is considered a key indicators of osteoblast induction [49]. The expression of *ALPL* increasing the induction time in the current study. However, due to the reduced activity of the induced cells,gene expression was not observed over 30 days. As the understanding of signaling pathways has expanded, an increasing number of scholars have used signaling pathway inhibitors to investigate the importance of pathways [50, 51]. We selected WNT antagonistI as a blocker of the *WNT* signaling pathway according to the literature [52, 53] and determined the optimal concentration to improve the results.

## Conclusions

The body size phenotype is the most direct manifestation of phenotypic differences among animals and is also a key characteristic used to identify livestock breeds. Our experiments showed that the important genes *M-Ras* and *ATF3* in the *GHR* signaling pathway were downregulated in the DP, indicating that changes in this pathway may drive important functions in this breed. In addition, the expression of *PLCɡ2* in the *WNT* signaling pathway was increased, which could increase Ca^2+^ export from cells through the transporters *TRPV4* and *SLC8A3* on the cell membrane. In addition, *ECM, ALPL, IHH, MMP23, TIMP4, COL10A1, Sox9, Sox6, Sox8, Sox11, COL2A1, COL9A1* and other factors were found to promote the early occurrence of biomineralization and epiphyseal closure in juvenile DPs. These two pathways may mediate the development of short stature. The purpose of this study was to reveal the molecular mechanism of short stature by analyzing the difference in body height between DPs and MHs. Our findings provide insight into the genetic regulation of growth short stature in mammals and can be used as a reference for the development of therapeutic strategies for small size. However, the association of these signaling pathways with body size traits in ponies needs further validation.

## Methods

### Collection of animal tissue samples

Animals were killed at the slaughterhouse, and we collected the tissues for our study. The health of all animals included in the study, was assessed by local veterinarians. Pituitary glands and the ends of long bones were obtained from six heathy female DPs and six heathy female MHs (three juveniles and three adults per breed) for transcriptomic analysis. In addition, liver, pituitary gland and long bone epiphyseal tissues were obtained from six healthy female DPs and six healthy female MHs (three juveniles and three adults per breed) for q-PCR and Western blot analysis. Whole blood samples and plasma samples were collected from ten healthy DPs and ten healthy MHs (five juveniles and five adults per breed) for SNP detection and hormone content determination. All tissues and samples were immediately snap frozen in liquid nitrogen and stored at − 80 °C until further use.

### RNA extraction

Each sample was individually ground (with a mortar and pestle under continuous liquid N^2^ chilling) into a fine powder before RNA extraction. Samples were stored at − 80 °C. Total RNA was extracted from 30 mg of tissue by using the hot phenol method. In brief, cell pellets were resuspended and washed once in Buffer A (50 mM sodium acetate and 10 mM EDTA, pH = 5.2). After collecting the cells by centrifugation, the pellets were resuspended in Buffer A containing 1% SDS and immediately added to hot phenol. After incubation at 65 °C for 5 min and centrifugation for 10 min at 4 °C, the RNA-containing supernatants were transferred to a new tube for ethanol precipitation, washed and dissolved in DEPC-treated water. The RNA was further purified with two phenol-chloroformextraction extraction steps and was then treated with RQ1 DNase (Promega) to remove DNA. The quality and quantity of the purified RNA were determined by measuring the absorbance ratio at 260 nm/280 nm (A260/A280) using a SmartSpec Plus (Bio-Rad). The integrity of the RNA was further verified by 1.5% agarose gel electrophoresis [54].

Ribosomal RNA was removed from the RNA samples (10 μg) using a RiboMinus rRNA Depletion Kit (Ambion), and the resulting samples were used to prepare directional RNA-seq libraries. The purified mRNA was then iron-fragmented at 95 °C before being subjected to end repair and 5′ adaptor ligation. Then, reverse transcription (RT) was performed using RT primers containing a 3′ adaptor sequence and a randomized hexamers cDNA was purified and amplified, and all 200–500 bp PCR products were purified, quantified and stored at − 80 °C until they were used for sequencing.

### Processing of raw RNA-seq data and evaluation and alignment of clean data

For high-throughput sequencing, libraries were constructed following the manufacturer’s instructions, and an Illumina GAIIx system was used to collect data via 151 bp single-end sequencing (ABlife Inc., Wuhan, China) [55]. Read quality was evaluated using FastQC [56]. Any reads less than 30 bp in length were removed using Btrim (with a parameter setting of-l = 30) [57], and the remaining reads were used for further analysis. The reads were mapped to the horse genome (EquCab 3.0). The Salmon software (version 1.1.0) was used to map the reads to the reference cDNA sequences and calculate the transcript per million mapped reads value of each transcript using the quasi-mapping method [58].

### Analysis of DEGs

To determine whether a gene was differentially expressed, we used the following thresholds: fold change (FC) > 2 or (FC) < − 2 and *P*-value (P) < 0.05. The *P*-value for differential expression was calculated in the R environment (version 3.6.3, https://www.r-project.org/) using the EdgeR package [59] (version 3.28.1), because it has good performance in the identification of DEGs from using biological triplicates [60]. EdgeR was downloaded from the Bioconductor website (www.bioconductor.org). To predict gene function and calculate the functional category distribution frequency, Kyoto Encyclopedia of Genes and Genomes (KEGG) and Gene Ontology (GO) analyses were employed using the Database for Annotation, Visualization and Integrated Discovery (DAVID) bioinformatics resource.

### Validation by q-PCR

In this study, q-PCR was performed on *GHR* and *GH* to validate the validity of the RNA-seq data. The expression was normalized to that of the reference gene *GAPDH* [61]*.* The primers are described in Additional files 13 and 14. The same RNA samples used for RNA-seq were used for q-PCR. One microgram of RNA was reverse transcribed using a Prime Script™ RT Reagent Kit (Takara) following the manufacturer’s instructions. q-PCR was performed in a Bio-Rad S1000 with Bestar SYBR Green RT-PCR Master Mix (DBI Bioscience) [62].

### Statistical analysis

All data were analyzed using SPSS 20.0 (SPSS, Inc., Chicago, USA). For relative quantitation, the F = 2^-ΔΔCt^ method was used, where 2-ΔΔCt reflected the relative expression level of the target gene in each sample relative to that in the control group. The remaining observations were paired; thus, a paired samples t-test was performed. *P* < 0.05 was considered to indicate statistically significance.

### Construction of an RNAi lentiviral vector

Based on the *GHR* gene sequence, we designed a sequence targeting the *GHR* gene for RNAi: 5 ‘- GCTGCAAGAATTGCTCATGAA − 3’. The GV493 vector (frame structure: hU6-CBh-gcGFP-IRES-puromycin, Shanghai Genechem Co., Ltd.) was used to construct the lentiviral vectors GV493-GHR-RNAi-a and GV493-GHR-RNAi-b. Single-stranded primers containing AgeI and EcoRI restriction sites were synthesized. Double-stranded DNA was generated by primer annealing. T4 DNA ligase was used to ligate the double-restriction-site target vector, and the double-stranded DNA was annealed. Competent cells were then transformed and positive bacterial colonies were identified via PCR [63]. Plasmid extraction and sequencing were carried out and the qualified plasmids were used in the follow-up experiment.

### RNAi lentiviral packaging

ATDC5 cells were treated 24 h before transduction. The cell density was adjusted to 5 × 10^6^cells/15 ml, and cells were cultured in a 10 cm cell culture dish at 37 °C in 5% CO_2_. Cells were transduced at 70–80% confluence. Serum-free medium was added 2 h before transfection. The prepared DNA solution (GV vector plasmid, 20 μg; pHelper1.0 vector plasmid, 15 μg; pHelper2.0 vector plasmid, 10 μg) and transduction reagent (Shanghai Genechem Co., Ltd.) were added to centrifuge tubes to a total volume of 1 ml. The centrifuge tubes were incubated at room temperature for 15 min, and the transduction mixtures were then added to ATDC5 cells and cultured for 6 h. The culture medium containing the transfection mixture was then dicarded, and the cells were washed with 10 ml of phosphate-buffered saline (PBS). Then, 20 ml of serum was added to the cells, which were then cultured for 48–72 h. The ATDC5 cell supernatant was collected and centrifuged at 4 °C and 4000×g for 10 min. The supernatant was filtered through a 0.45 μm filter and centrifuged at 4 °C and 25,000 r/min for 2 h and discarded. Virus preservation solution was added to resuspend the pellet and the solution was centrifuged at 10000 r/min for 5 min. The supernatant was subpacked, and ATDC5 cells were cultured in 96-well plates (4 × 10^4^ cells/well, 100 μl). Gradient dilutions of lentiviral particles were added, and cells were cultured for 24 h. Then complete medium was added. The expression of fluorescent protein was observed after 4 days and the viral titer was calculated [64].

### Lentiviral transduction

ATDC5 cells were subcultured at 80% confluence in 6-well plates (3–5 × 10^4^ cells/well, 2 ml). ATDC5 cells at 20% confluence were infected with lentivirus. The cells were then divided into two groups: a negative control group and an RNAi lentivirus-infected group. Transduction was performed with and without puromycin. The culture medium was replaced after 16 h and the cells were photographed after 72 h [65].

### Apoptosis analysis

Cells were treated with apoptosis assay kit resgents (eBioscience 88–8007), washed with ice-cold PBS, resuspended in binding buffer, and then incubated with Annexin V-APC for 10 min at room temperature in the dark. Finally, the apoptotic cells were analyzed by a FACS (Becton-Dickinson, USA).

### Isolation and culture of horse BMSCs

BMSCs originally isolated from MHs were used in our laboratory at passage 3 (P3). After adult horses were slaughtered, the sternum was removed and sterilized with 75% alcohol. The following procedures were performed under sterile conditions. Two ends of the sternum were washed out with medium containing 1% penicillin and streptomycin in PBS, and the wash fluid was collected in 15 ml sterile centrifuge tubes for centrifugation of at 1500 r/min for 5 min. The supernatant was suspended in complete medium (DMEM/F-12 containing, 15% FBS, 0.1% penicillin, and 0.1% streptomycin) and inoculated in a flask at a density of 5 × 10^6^cells. The supernatant was cultured in an incubator at 37 °C, in 5% CO_2_ and 100% humidity. After 48 h, half of the supernatant was replaced. At 70–80% confluence, the cells were digested with trypsin and passaged at a ratio of 1:3.

### Induction of horse BMSCs differentiation into osteoblasts

BMSCs at P3 cells were divided into an induction group and a noninduction group. The cells were 70–80% confluent, induction medium (osteogenic induction medium: 0.1 mM dexamethasone, 10 mM beta-glycerophosphate disodium hydrate, 50 mg/l Vc) was added to the induction group. Cells were cultured for 28 days, and the medium was replaced every other day. The control undifferentiated group continued to be cultured in a general culture medium, and the cell state was photographed and noted daily.

### Alizarin red and Alcian blue staining

After 7, 14 and 21 days of culture, Alizarin red and Alcian blue staining were performed on the induced and noninduced cells, respectively. When the cells were 70–80% confluent, they were washed with PBS 3 times for 2 min each and were then fixed with 4% paraformaldehyde at room temperature for 30 min. The cells were then washed with PBS 3 times for 2 min each stained with solution for 5 min and washed again with PBS 3 times for 2 min each. Finally, the cells were visualized under the micoscope.

### MTT assay with ATDC5 cells and osteoblasts

An MTT assay was used to evaluate cell proliferation. The mouse chondroprogenitor cell line ATDC5 was obtained from TongPai (Shanghai) Biotechnology Co., Ltd. Cells were cultured in a 96-well plate at a density of 1 × 10^5^ cells/ml and incubated in complete medium at 37 °C. After 24 h, WNT antagonistIwas added to in serum-free medium at different concentrations, with 3 replicates per concentration. After 4 h of incubation, the medium was replaced with PBS, and 20 μl of 20 mM MTT was added and incubated for 3 h at 37 °C. After 3 h, DMSO was added to dissolve the purple formazan crystals. The cell plates were placed on a horizontal shaker for 5 min to complete dissolution. The optical density was measured in an enzyme-linked immunoassay reader at an excitation wavelength of 490 nm [63]. Analyses were performed in triplicate.

## Supplementary Information

**Additional file 1:.** Heights of the Debao pony and Mongolian horse at two developmental stages.

**Additional file 2:.** Sample correlation between tissues of Debao ponies and tissues of Mongolian horses.

**Additional file 3:.** Expression levels of GH in the pituitaries of Debao ponies and Mongolian horses.

**Additional file 4:.** Expression levels of GHR in the long bones of Debao ponies and Mongolian horses.

**Additional file 5:.** Expression of the GH protein in the pituitaries and long bones of Debao ponies and Mongolian horses.

**Additional file 6:.** Expression levels of IGF-1 in the livers of Debao ponies and Mongolian horses.

**Additional file 7:.** Easy-siRNA design; Infection was performed at different MOIs. According to the efficiency of cell transduction, an MOI with a high infection efficiency that yielded a good cell status was selected. In ATDC5 cells at 70–80% confluence, optimal infection was achieved with HiTransG P and an MOI of approximately 10.

**Additional file 8:.** GHR-RNAi expression in ATDC5 cells.

**Additional file 9:.** Detection of apoptosis by annexin V-APC staining.

**Additional file 10:.** Primary culture of horse BMSCs; electrophoresis of osteoblast marker genes, differentiation of osteoblasts, and Alizarin red (a and b) and Alcian blue (c and d) staining of horse induced BMSCs at day 28.

**Additional file 11:.** Induction of the expression of cartilage-specific genes on different days.

**Additional file 12:.** Primer sequences used for amplification of horse genes (PCR).

**Additional file 13:.** Primer sequences used for amplification of horse genes (q-PCR).

**Additional file 14:.** Primer sequences used for amplification of mouse genes (q-PCR).

## Data Availability

The data discussed in this publication have been deposited in the National Center for Biotechnology Information (NCBI) Sequence Read Archive and are accessible under GEO Series Accession No. GSE146145. We confirm that all experiments were carried out in accordance with the relevant guidelines and regulations.

## Abbreviations

ALP: Alkaline phosphatase
ATF3: Activating transcription factor 3
ADAMTS17: ADAM metallopeptidase with thrombospondin type 1 motif 17
BMSCs: Horse bone marrow mesenchymal stem cells
BMP: Bone morphogenetic protein
DP: Debao pony
DPPJ: Juvenile Debao pony pituitary
DPPA: Adult Debao pony pituitary
DPLBJ: Juvenile Debao pony long bone
DPLBA: Adult Debao pony long bone
CAMKII: Calcium/calmodulin dependent protein kinase II
COL6A1: Collagen type VI alpha 1 chain
COL2A1: Collagen type II alpha 1 chain
COL9A1: Collagen type IX alpha 1 chain
CGA: Glycoprotein hormones, alpha polypeptide
ECM: Extracellular matrix
EGR1: Early growth response 1
DKC1: Dyskerin pseudouridine synthase 1
FZD2: Frizzled class receptor 2
GH: Growth hormone
GHR: Growth hormone receptor
GHRH: Growth hormone releasing hormone.
HMGA2: High mobility group AT-hook 2
IGF-1: Insulin-like growth factors-1
ITPKC: Inositol trisphosphate 3 kinase C
IL8: Interleukin 8
JAK2: Janus kinase 2
MH: Mongolian horse
MHPJ: Juvenile Mongolian horse pituitary
MHPA: Adult Mongolian horse pituitary
MHLBJ: Juvenile Mongolian horse long bone
MHLBA: Adult Debao pony long bone
MMP: Matrix metalloproteinase
MYD88: MYD88 innate immune signal transduction adaptor
MAPK: Mitogen-activated protein kinase
m-RAS: Muscle RAS oncogene homolog
OSTN: Osteocrin
PLCβ2: Phospholipase C beta 2
PLCg2: Phospholipase C gamma 2
qRT-PCR: Quantitative real time polymerase chain reaction
RUNX2: Runt-related transcription factor 2
SNP: Single-nucleotide polymorphism
SMAD2: SMAD family member 2
STC2: Stanniocalcin 2
SMS: Spermine synthase
SLC8A3: Solute carrier family 8 member A3
SOCS1: Suppressor of cytokine signaling 1
TRPV4: Transient receptor potential cation channel subfamily V member 4
TSHR: Thyroid stimulating hormone receptor
TIMP4: Tissue inhibitor of metallopeptidases 4
TNFRSF: Tumornecrosis factor receptor superfamily
TSH: Thyroid stlmulating hormone
TGFβ3: Transforming growth factor beta 3
TLR2: Toll like receptor 2
TBX3: T-box transcription factor 3
WNT5A: WNT family member 5A
