# Supplementary material for: Pathways involved in pony body size development
Source: BMC Genomics. 2021 Jan 18;22:58. doi: 10.1186/s12864-020-07323-1 (PMC7814589; doi:10.1186/s12864-020-07323-1)
Supplement: Supplementary file 1 — Additional file 1:. Heights of the Debao pony and Mongolian horse at two developmental stages. [file 12864_2020_7323_MOESM1_ESM.docx]

**Additional file 1.**


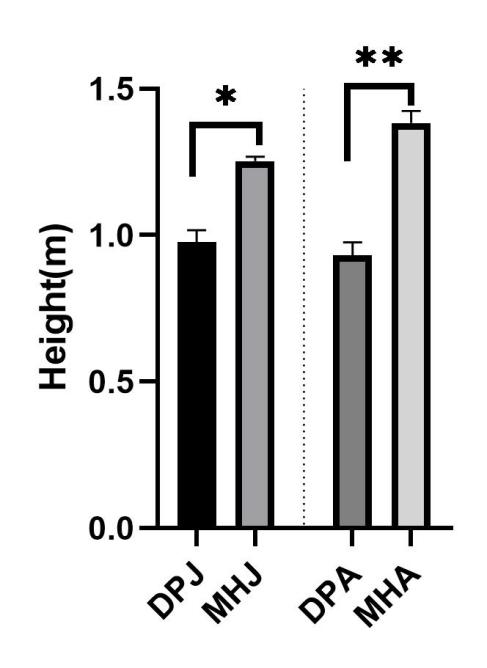


Heights of the Debao pony and Mongolian horse at two developmental stages.

Note: DPA, Debao pony at the adult stage; DPJ, Debao pony in the juvenile stage, MHA, Mongolian horse at the adult stage; MHJ, Mongolian horse in the juvenile stage.
