# Supplementary material for: Pathways involved in pony body size development
Source: BMC Genomics. 2021 Jan 18;22:58. doi: 10.1186/s12864-020-07323-1 (PMC7814589; doi:10.1186/s12864-020-07323-1)
Supplement: Supplementary file 2 — Additional file 2:. Sample correlation between tissues of Debao ponies and tissues of Mongolian horses. [file 12864_2020_7323_MOESM2_ESM.docx]

**Additional file 2.**


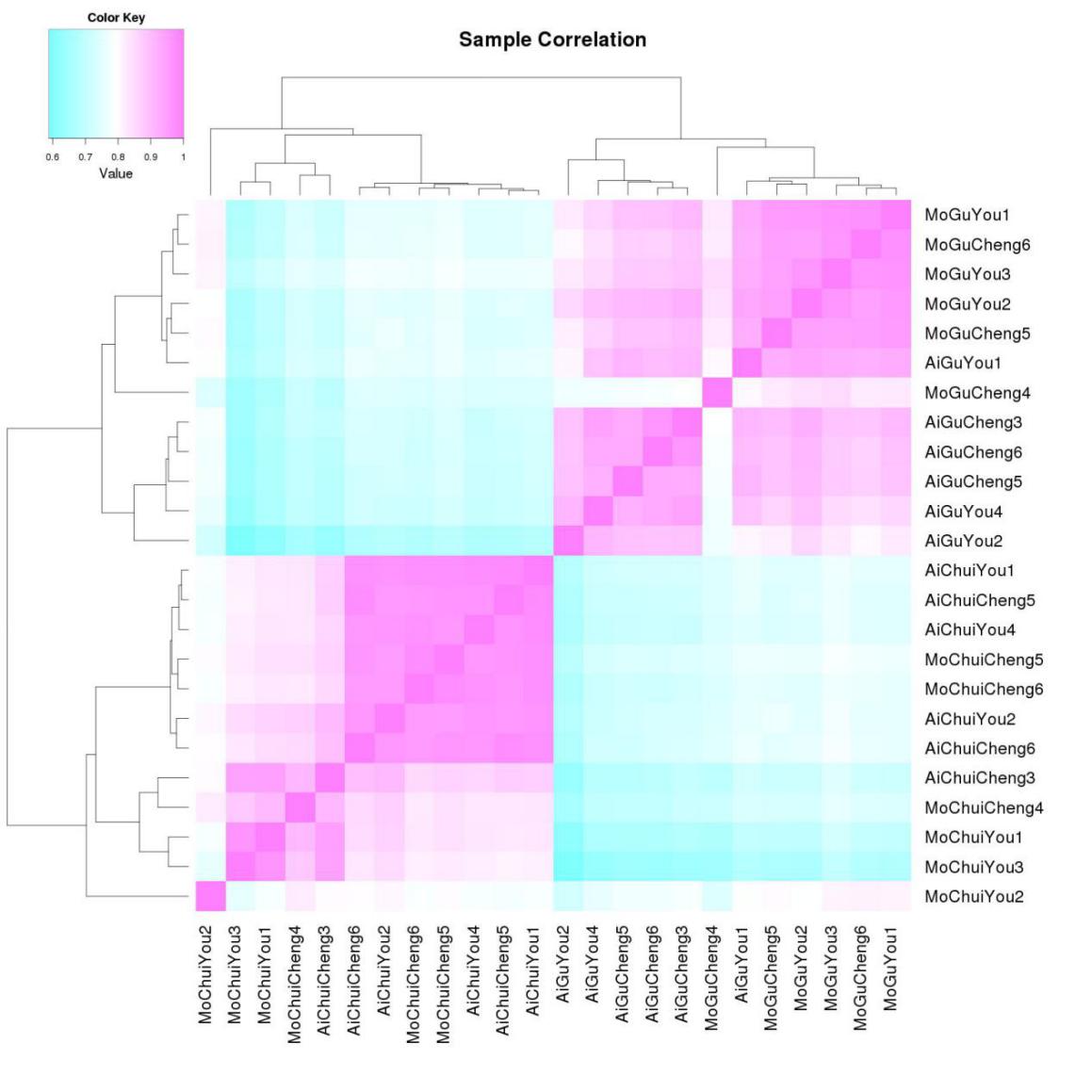


Sample correlation between tissues of Debao ponies and Mongolian horses.

Note: AiChuiCheng represents the pituitary gland of Debao ponies at the adult stage, AiChuiYou represents the pituitary gland of Debao ponies at the juvenile stage, MoChuiCheng represents the pituitary gland of Mongolian horses at the adult stage, and MoChuiYou represents the pituitary gland of Mongolian horses at the juvenile stage. AiGuCheng represents the epiphysis of Debao ponies at the adult stage, AiGuYou represents the epiphysis of Debao ponies at the juvenile stage, MoGuCheng represents the epiphysis of Mongolian horses at the adult stage, and MoGuYou represents the epiphysis of Mongolian horses at the juvenile stage.
