# Supplementary material for: Pathways involved in pony body size development
Source: BMC Genomics. 2021 Jan 18;22:58. doi: 10.1186/s12864-020-07323-1 (PMC7814589; doi:10.1186/s12864-020-07323-1)
Supplement: Supplementary file 3 — Additional file 3:. Expression levels of GH in the pituitaries of Debao ponies and Mongolian horses. [file 12864_2020_7323_MOESM3_ESM.docx]

**Additional file 3.**


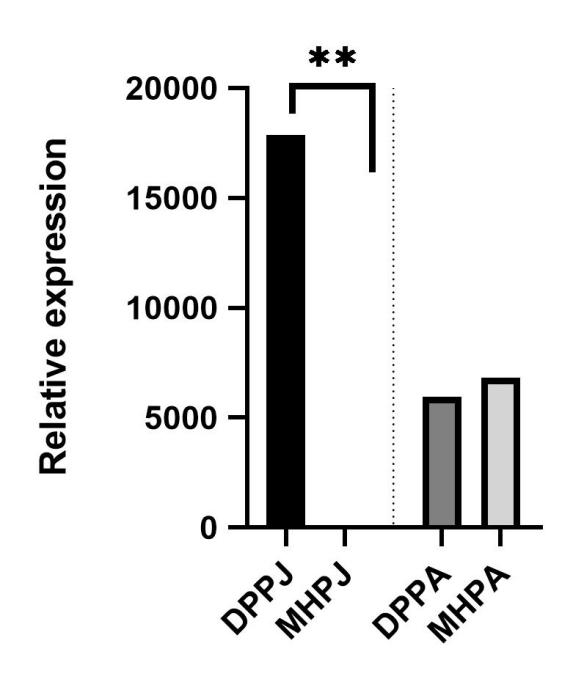


Expression levels of *GH* in the pituitaries from Debao ponies and Mongolian horses.

Note: MHPJ, juvenile Mongolian horse pituitary; MHPA, adult Mongolian horse pituitary; DPPJ, juvenile Debao pony pituitary; DPPA, adult Debao pony pituitary.
