# Supplementary material for: Pathways involved in pony body size development
Source: BMC Genomics. 2021 Jan 18;22:58. doi: 10.1186/s12864-020-07323-1 (PMC7814589; doi:10.1186/s12864-020-07323-1)
Supplement: Supplementary file 4 — Additional file 4:. Expression levels of GHR in the long bones of Debao ponies and Mongolian horses. [file 12864_2020_7323_MOESM4_ESM.docx]

**Additional file 4.**


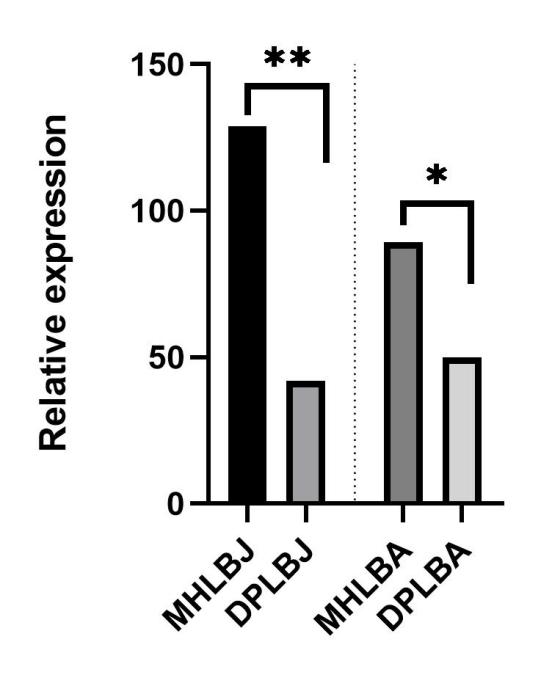


Expression levels of *GHR* in the long bones of Debao ponies and Mongolian horses.

Note: MHLBJ, juvenile Mongolian horse long bones; MHLBA, adult Mongolian horse long bones; DPLBJ, juvenile Debao pony long bones; DPLBA, adult Debao pony long bones.
