# Supplementary material for: Pathways involved in pony body size development
Source: BMC Genomics. 2021 Jan 18;22:58. doi: 10.1186/s12864-020-07323-1 (PMC7814589; doi:10.1186/s12864-020-07323-1)
Supplement: Supplementary file 5 — Additional file 5:. Expression of the GH protein in the pituitaries and long bones of Debao ponies and Mongolian horses. [file 12864_2020_7323_MOESM5_ESM.docx]

**Additional file 5.**


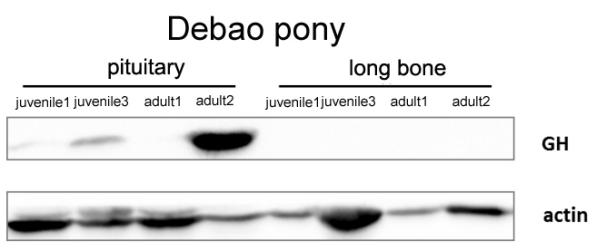


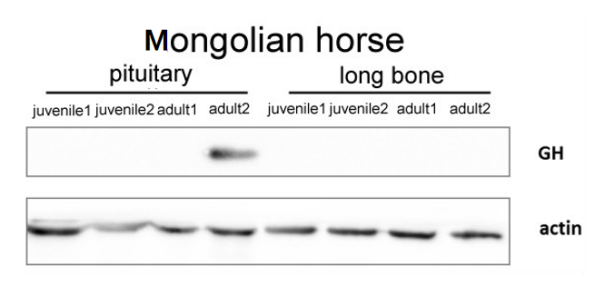


Expression of the GH protein in the pituitaries and long bones of Debao ponies and Mongolian horses.
