# Supplementary figures and images for: Pathways involved in pony body size development
Source: BMC Genomics. 2021 Jan 18;22:58. doi: 10.1186/s12864-020-07323-1 (PMC7814589; doi:10.1186/s12864-020-07323-1)

**Additional file 6.**


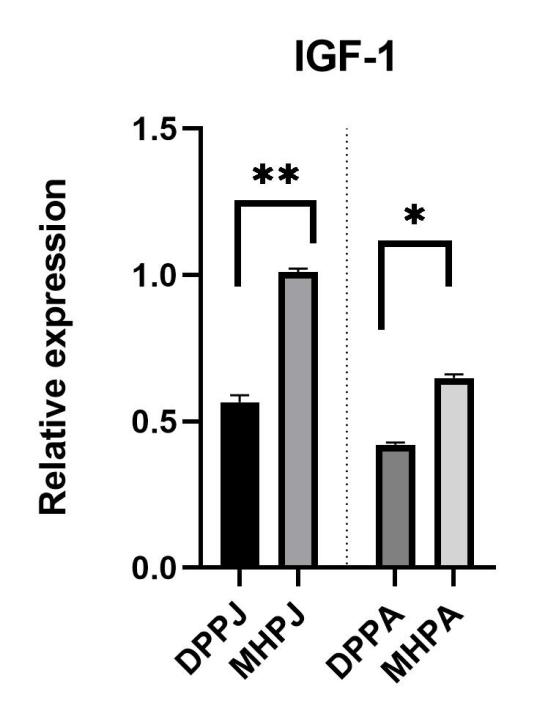


Expression levels of IGF-1 in the liver of Debao ponies and Mongolian horses.

Supplement: Supplementary file 6 — Additional file 6:. Expression levels of IGF-1 in the livers of Debao ponies and Mongolian horses. [file 12864_2020_7323_MOESM6_ESM.docx]

**Additional file 8.**


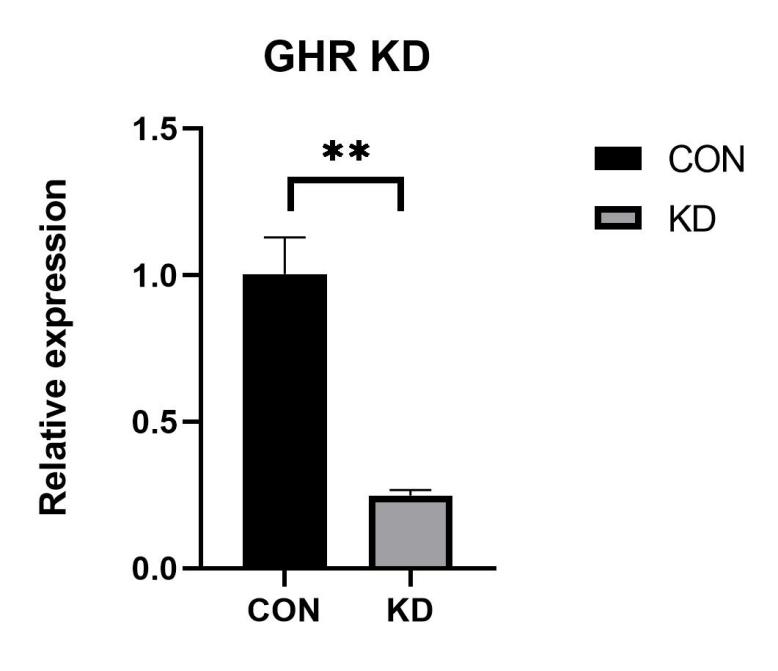


GHR-RNAi expression in ATDC5 cells.

Supplement: Supplementary file 8 — Additional file 8:. GHR-RNAi expression in ATDC5 cells. [file 12864_2020_7323_MOESM8_ESM.docx]

**Additional file 11.**


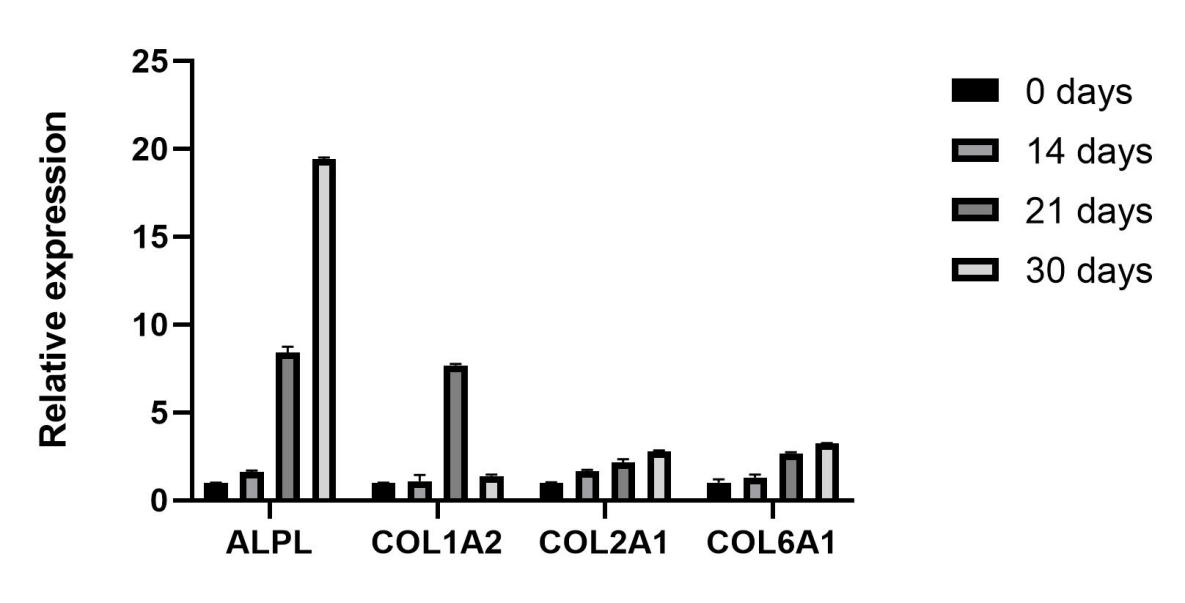


Induction of cartilage-specific gene expression on different days.

Supplement: Supplementary file 11 — Additional file 11:. Induction of the expression of cartilage-specific genes on different days. [file 12864_2020_7323_MOESM11_ESM.docx]
