# Supplementary material for: Pathways involved in pony body size development
Source: BMC Genomics. 2021 Jan 18;22:58. doi: 10.1186/s12864-020-07323-1 (PMC7814589; doi:10.1186/s12864-020-07323-1)
Supplement: Supplementary file 7 — Additional file 7:. Easy-siRNA design; Infection was performed at different MOIs. According to the efficiency of cell transduction, an MOI with a high infection efficiency that yielded a good cell status was selected. In ATDC5 cells at 70–80% confluence, optimal infection was achieved with HiTransG P and an MOI of approximately 10. [file 12864_2020_7323_MOESM7_ESM.docx]

**Additional file 7.**

Easy-siRNA design.

| No. | Accession | Target Seq | CDS | GC% |
| --- | --- | --- | --- | --- |
| Ghr-RNAi (74225-1) | NM_001048178 | GCTGCAAGAATTGCTCATGAA | 363..1256 | 42.86% |
| Description | *Mus musculus* growth hormone receptor (GHR), transcript variant 2, mRNA | | | |

1. ］
2. ］


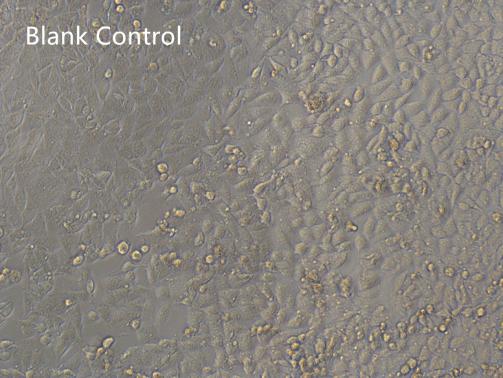

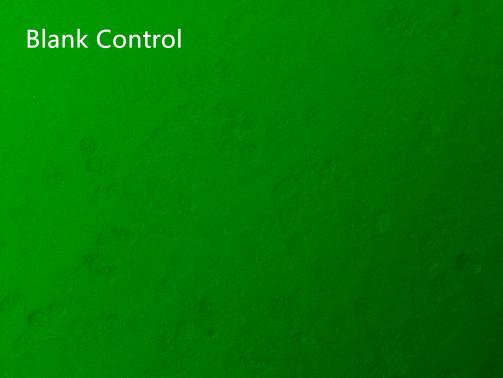

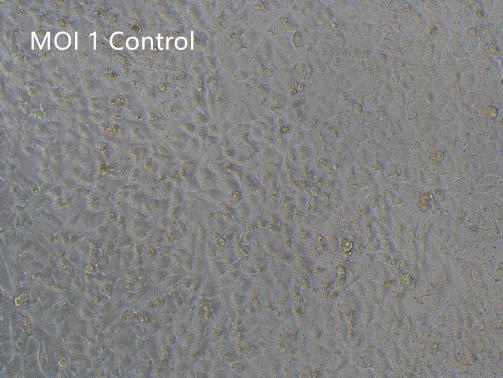

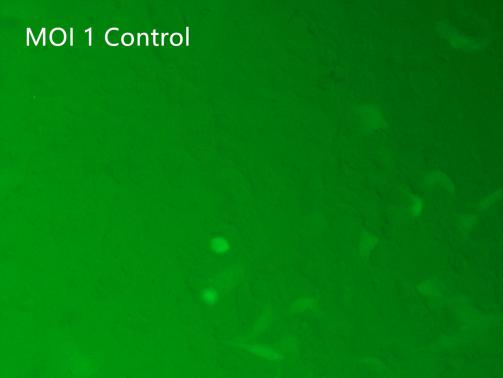

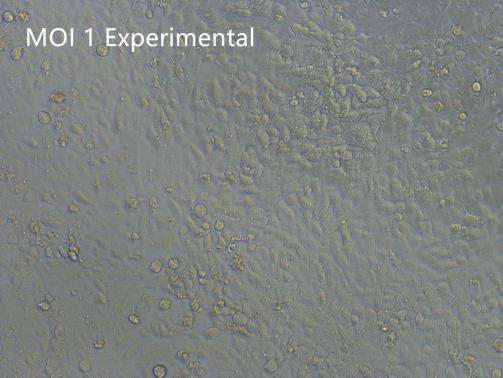

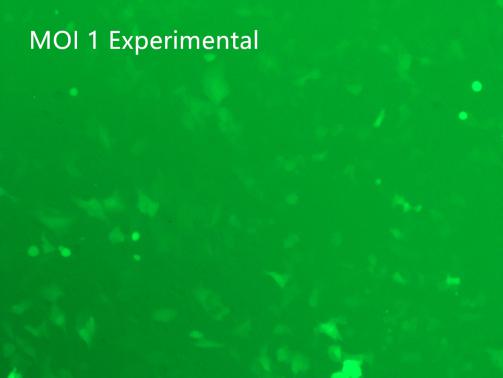

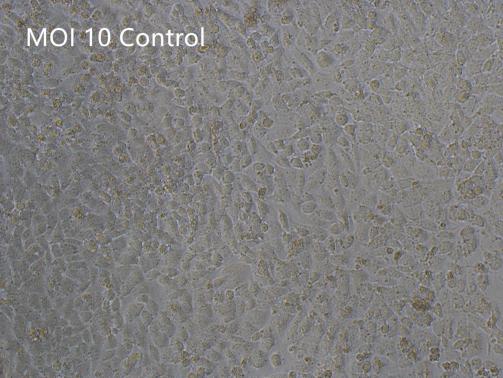

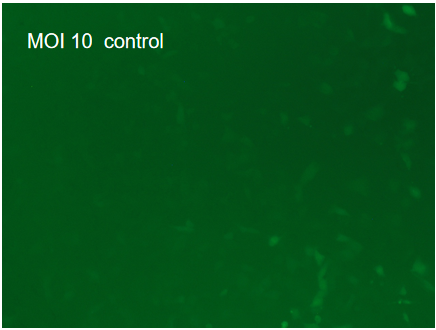


1. ］

20 µm

1. ］

20 µm

1. ］

20 µm

1. ］

20 µm

1. ］

20 µm

1. ］

20 µm

1. ］

20 µm

1. ］

20 µm


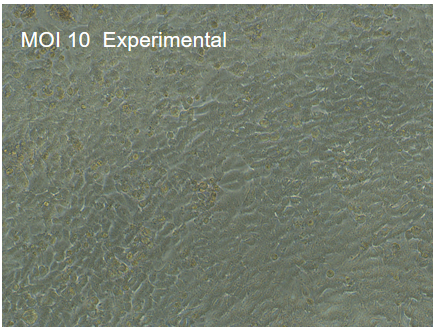

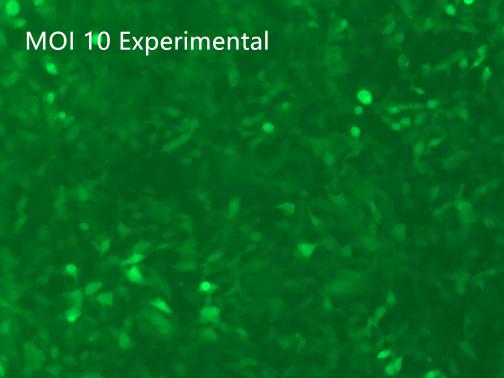

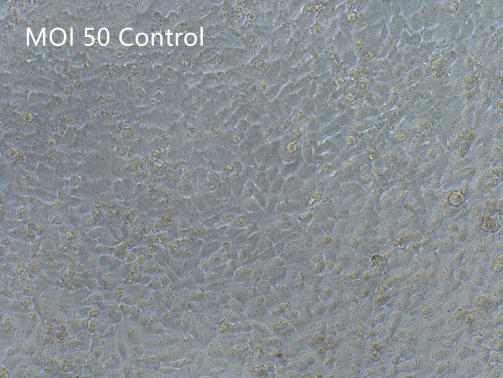

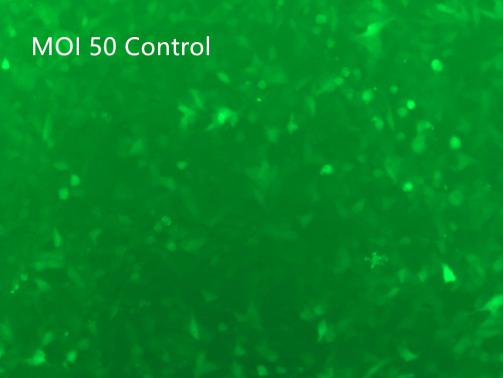

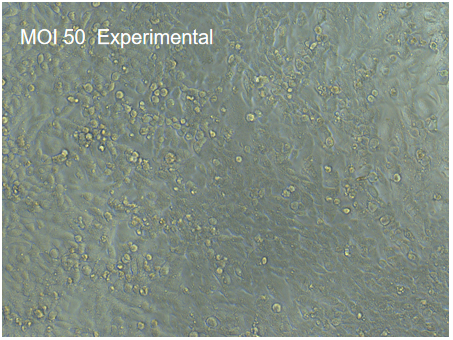

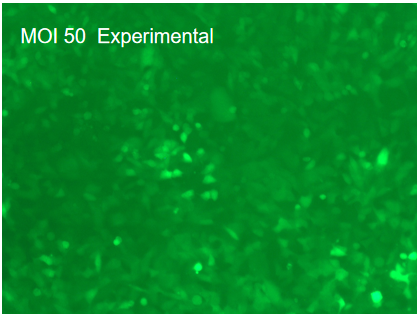

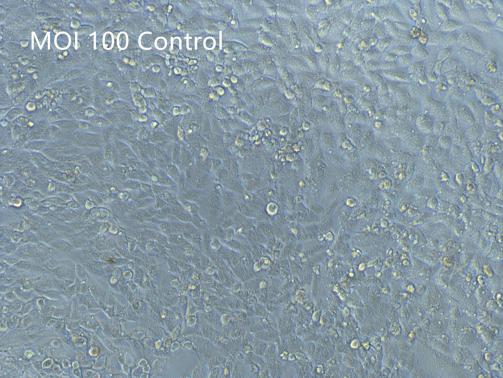

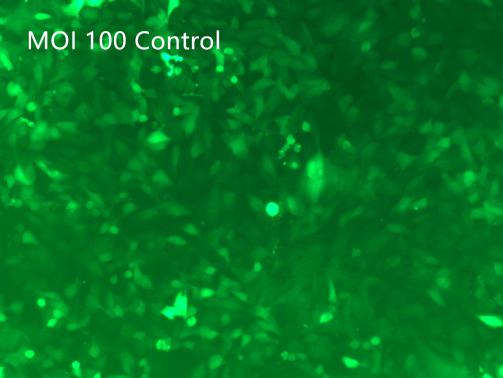


1. ］

20 µm

1. ］

20 µm

1. ］

20 µm

1. ］

20 µm

1. ］

20 µm

1. ］

20 µm

1. ］

20 µm

1. ］

20 µm


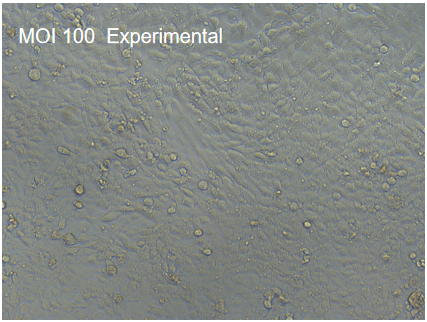

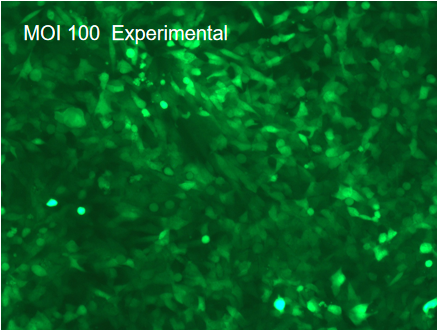


1. ］

20 µm

1. ］

20 µm

Infection at different MOIs (100×)

Note: According to the efficiency of cell transduction, an MOI with a high infection efficiency and good cell status was selected.The viral titer at different MOIs was compared between the control group (ATDC5) and the experimental group (KD-ATDC5). In ATDC5 cells at 70-80% confluence, optimal infection was achieved with HiTransG P and an MOI of approximately 10.
