# Supplementary material for: Pathways involved in pony body size development
Source: BMC Genomics. 2021 Jan 18;22:58. doi: 10.1186/s12864-020-07323-1 (PMC7814589; doi:10.1186/s12864-020-07323-1)
Supplement: Supplementary file 9 — Additional file 9:. Detection of apoptosis by annexin V-APC staining. [file 12864_2020_7323_MOESM9_ESM.docx]

**Additional file 9.**


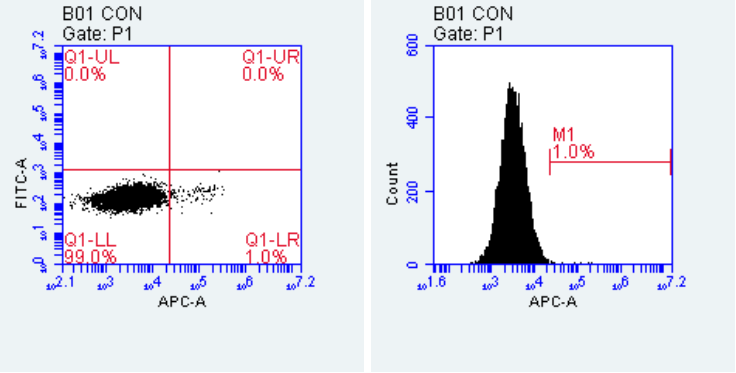

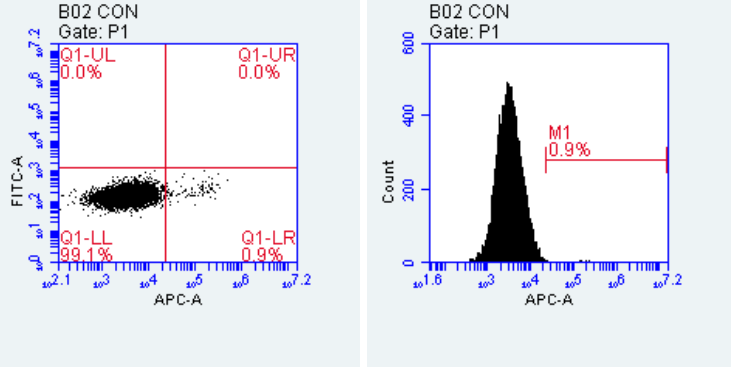


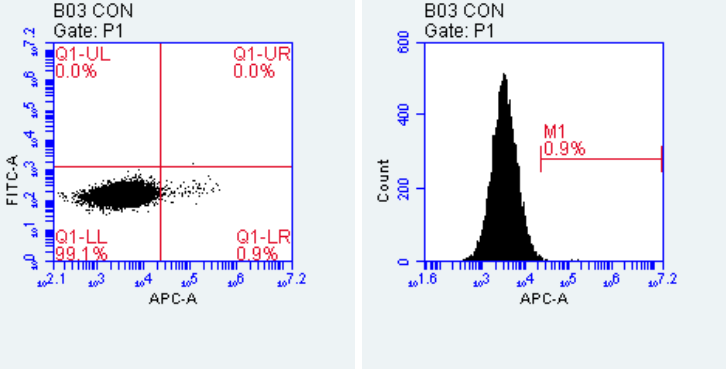

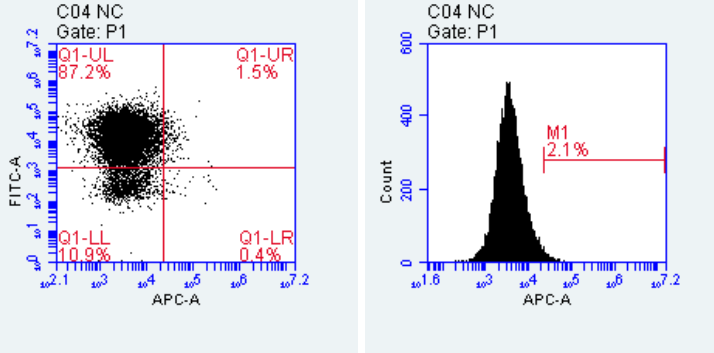


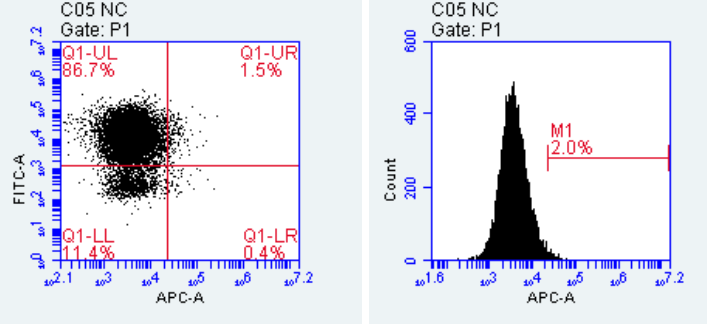

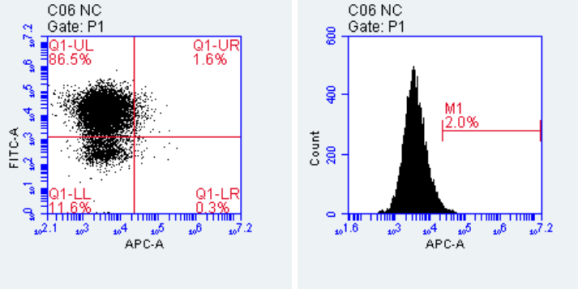


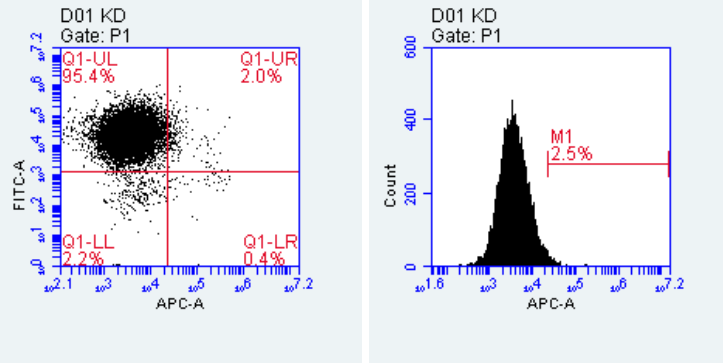

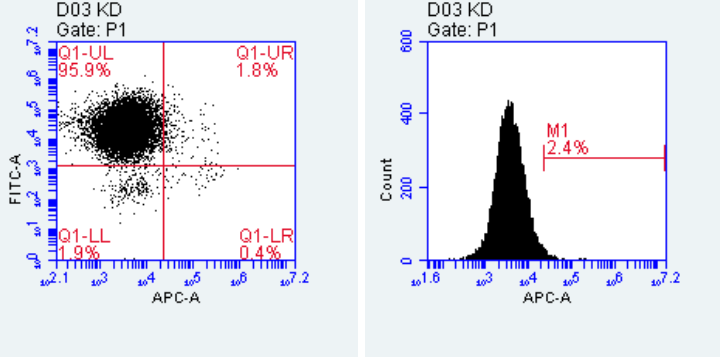


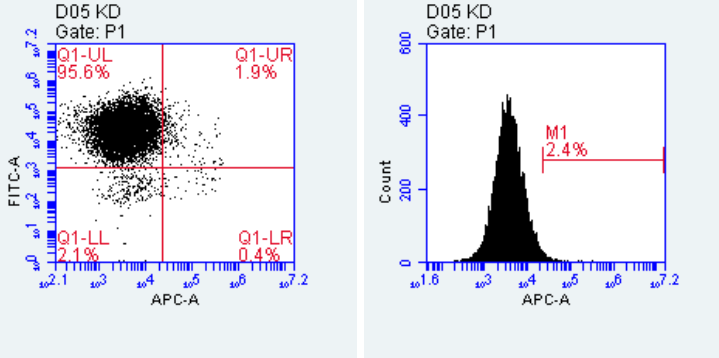


Detection of apoptosis by annexin V-APC staining.

Note: In this experiment, a single staining apoptosis assay was carried out, including ATDC5 blank cells (B01, B02, B03), negative control (NC) cells (C04, C05, C06) and knockdown (KD) cells (D01, D03, D05), with three replicates per group. No fluorescence was observed in the blanck cells. NC and KD cells were included in the virus group, and the virus expressed green flurescent protein. Any group of cells with early apoptosis would be stained red by the kit. For the four quadrant diagram: the lower left corner represents the cells that did not undergo apoptosis or virus fluorescence; the upper left corner represents the group of virus-infection cells that exhibited green fluorescence, but did not undergo apoptosis; the upper right corner represents the group of virus-infected and apoptotic cells; and the lower right corner represents the group of non-viral-infected but apoptotic cells.
