# Supplementary material for: Pathways involved in pony body size development
Source: BMC Genomics. 2021 Jan 18;22:58. doi: 10.1186/s12864-020-07323-1 (PMC7814589; doi:10.1186/s12864-020-07323-1)
Supplement: Supplementary file 10 — Additional file 10:. Primary culture of horse BMSCs; electrophoresis of osteoblast marker genes, differentiation of osteoblasts, and Alizarin red (a and b) and Alcian blue (c and d) staining of horse induced BMSCs at day 28. [file 12864_2020_7323_MOESM10_ESM.docx]

**Additional file 10.**


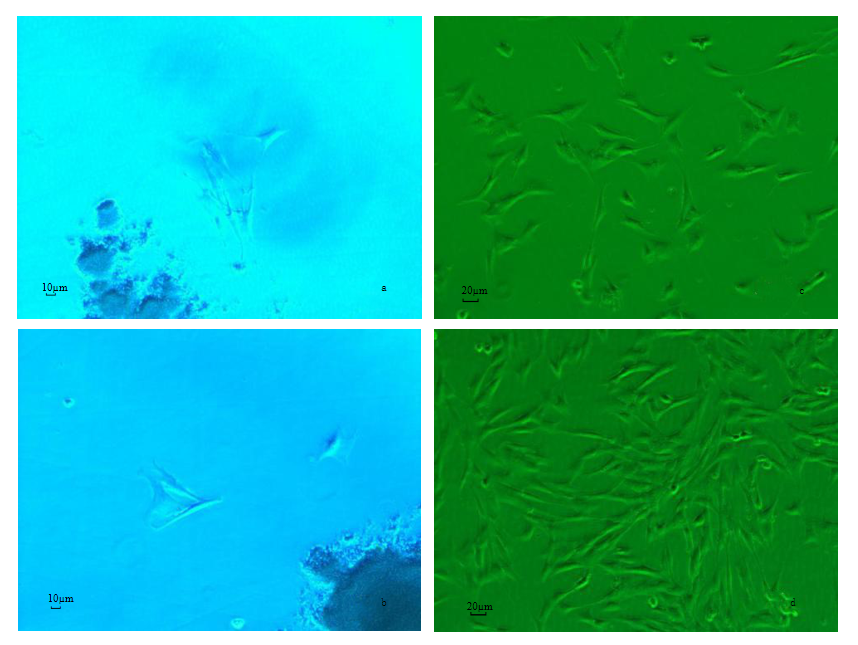


Primary culture of horse bone marrow mesenchymal stem cells (100×).

Note: a, b. Free cells cultured for 3 to 4 days; c, d. State of cells cultured for 7 to 8 days.


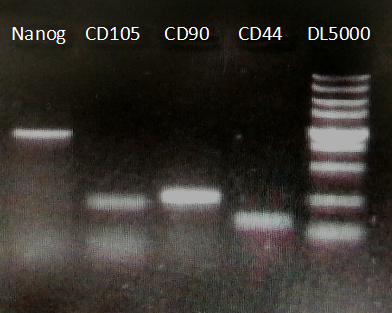


Electrophoresis of osteoblast marker genes.

Note: The lengths of the *CD34, Nanog, CD105, CD90* and *CD44* transcripts were 2724 bp, 2003 bp, 301 bp, 361 bp and 142 bp, respectively.

This picture has been cropped. The original picture is shown in the original document 2.


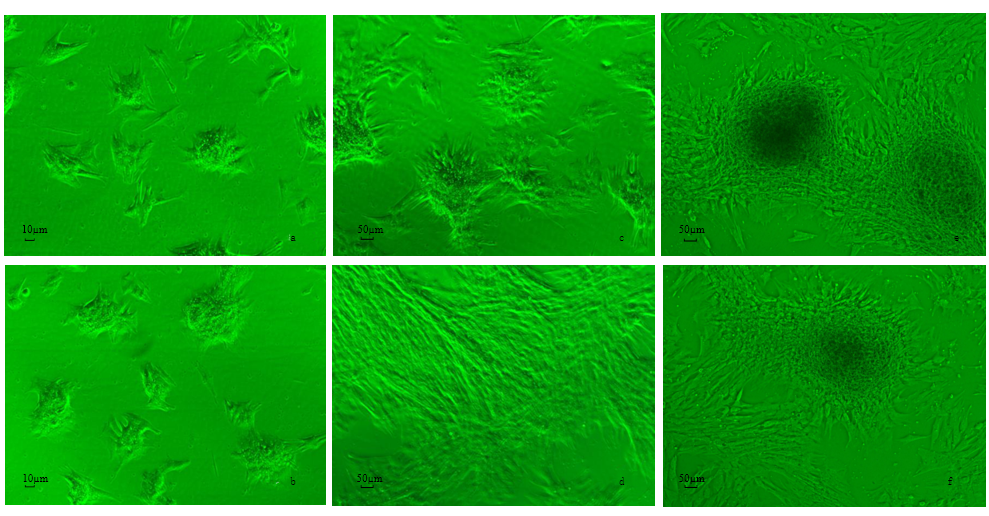


Differentiated osteoblasts (100×).

Note: a, b. Bone marrow mesenchymal stem cell morphology on the second day of differentiation induction; c, d. Bone marrow mesenchymal stem cell morphology on the 14th day of differentiation induction; e, f. Bone marrow mesenchymal stem cell morphology on the 28th day of differentiation induction.


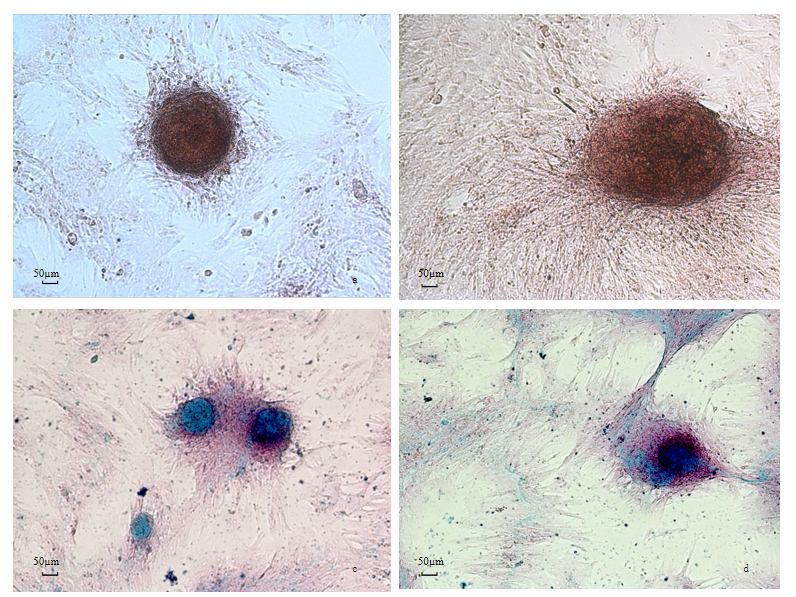


Alizarin red (a and b) and Alcian blue (c and d) staining of induced horse BMSCs on day 28.
