# Supplementary material for: Pathways involved in pony body size development
Source: BMC Genomics. 2021 Jan 18;22:58. doi: 10.1186/s12864-020-07323-1 (PMC7814589; doi:10.1186/s12864-020-07323-1)
Supplement: Supplementary file 12 — Additional file 12:. Primer sequences used for amplification of horse genes (PCR). [file 12864_2020_7323_MOESM12_ESM.docx]

**Additional file 12.**

Primer sequences used for amplification of horse genes (PCR).

| Gene | Primer Sequence (5’-3’) | Annealing temperature (℃) | Product size | GenBank accession number |
| --- | --- | --- | --- | --- |
| Nanog | F TACCTCAGCCTCCAGCAGATGC | 61 | 2003 bp | XM_023643093.1 |
|  | R CCAGGAATGGTTGCTCCAAGACTG |  |  |  |
| CD44 | F CCACTAATGCGAGTCACCACA | 57 | 142 bp | EU366979.1 |
|  | R TCATCATCATCAATGCCTGATCCA |  |  |  |
| CD90 | F CCATGAGAATACCACCGCCACAC | 61 | 361 bp | EU881920.1 |
|  | R GGAGCACAGAGACAGTCTTGTTGG |  |  |  |
| CD105 | F AGTCTGCATTGCACCGTCATGAG | 61 | 301 bp | KF181205.1 |
|  | R AGCGAGAGGCTCTCCGTGTTG |  |  |  |
