# Supplementary material for: Pathways involved in pony body size development
Source: BMC Genomics. 2021 Jan 18;22:58. doi: 10.1186/s12864-020-07323-1 (PMC7814589; doi:10.1186/s12864-020-07323-1)
Supplement: Supplementary file 13 — Additional file 13:. Primer sequences used for amplification of horse genes (q-PCR). [file 12864_2020_7323_MOESM13_ESM.docx]

**Additional file 13.**

Primer sequences used for amplification of horse genes (q-PCR).

| Gene | Primer Sequence (5’-3’) | | Annealing temperature (℃) | Product size | GenBank accession number |
| --- | --- | --- | --- | --- | --- |
| GAPDH | F | TTCAACGGCACAGTCAAG | 57 | 159 bp | XM_014734891.2 |
|  | R | TACTCAGCACCAGCATCA |  |  |  |
| WNT5A | F | GCGGCTACGACCAGTTCAAGAC | 61 | 183 bp | XM_02362026.1 |
|  | R | TGACAGGCGGACGACGAGAC |  |  |  |
| WNT2 | F | TCTCGGTGGAATCTGGCTCTGG | 61 | 151 bp | NM_001114150.1 |
|  | R | AATGGCACGCATCACGTCTGG |  |  |  |
| WNT4 | F | CGAGGAGGAGACCTGCGAGAAG | 61 | 190 bp | XM_023635552.1 |
|  | R | GCGTCACCACCTTGCCGAAG |  |  |  |
| WNT11 | F | CTGCCATCAGCCACGCCATC | 61 | 130 bp | XM_023645687 |
|  | R | GCTGAGGTTGTCCGCACATCC |  |  |  |
| PLCβ2 | F | TGTGGAGTGGTGGCTGGTCAG | 56 | 156 bp | XM_023620079.1 |
|  | R | ACGTCACTGCCGCACACTTAAC |  |  |  |
| PLCɡ2 | F | GCCAACCAGCAAGACCAAGGAC | 56 | 138 bp | XM_001501998 |
|  | R | ACACTGTCCGCCTTCGTCTCC |  |  |  |
| FZD22 | F | ATGAAGCACGACGGCACCAAG | 56 | 111 bp | NC_009154.3 |
|  | R | GAAGTAGCAGGCGATGACGATGG |  |  |  |
| CAMK2A | F | CCGTGGACTGCCTGAAGAAGTTC | 54 | 178 bp | XM_005599299 |
|  | R | TTCAATGGTGGTGTTGGTGCTCTC |  |  |  |
| ALPL | F | CCAGGCACAAGCACTCTCACTAC | 56 | 136 bp | XM_005607380.3 |
|  | R | TCGGTCACGTTGTTCCTGTTCAG |  |  |  |
| COL6A1 | F | GTACAAGGACGGAGCGGTCAATG | 56 | 90 bp | XM_001488351.5 |
|  | R | AGCGACACATCCAACAGGAACAC |  |  |  |
| COL1A2 | F | CCGTGGAAGCGATGGAAGTGTG | 57 | 349 bp | XM_001492939.4 |
|  | R | ACCAGGCTCACCAACGAGTCC |  |  |  |
| COL2A1 | F | GGAGCAGCAAGAGCAAGGACAAG | 56 | 138 bp | NM_001081764.1 |
|  | R | TGGACAGCAGACGCAGGAAGG |  |  |  |
