# Supplementary material for: Pathways involved in pony body size development
Source: BMC Genomics. 2021 Jan 18;22:58. doi: 10.1186/s12864-020-07323-1 (PMC7814589; doi:10.1186/s12864-020-07323-1)
Supplement: Supplementary file 14 — Additional file 14:. Primer sequences used for amplification of mouse genes (q-PCR). [file 12864_2020_7323_MOESM14_ESM.docx]

**Additional file 14.**

Primer sequences used for amplification of mouse genes (q-PCR).

| Gene | Primer Sequence (5’-3’) | | Annealing temperature (℃) | Product size | GenBank accession number |
| --- | --- | --- | --- | --- | --- |
| WNT5A | F | CATTGGAGAAGGTGCGAAGACAGG | 62 | 144 bp | XM_006518924.2 |
|  | R | GCTCGGCTCATGGCGTTCAC |  |  |  |
| WNT2 | F | TGCAAGTGTCATGGTGTGAGTGG | 61 | 184 bp | NM_023653.5 |
|  | R | CTACAGTGAAGCCAGTGCCATCC |  |  |  |
| WNT4 | F | GGTAATGATGCCTGTCAGCCTCAC | 61 | 197 bp | NM_009523.2 |
|  | R | CCTCTCCACTCAGCCTCTTAGCC |  |  |  |
| WNT11 | F | CGCCACCATCAGTCACACCATC | 61 | 128 bp | NM_0012857921 |
|  | R | GCTGAGGTTGTCCGCACATCC |  |  |  |
| PLCβ2 | F | CCGCATTGATGTGGTGGTAGCC | 56 | 89 bp | NM_177568.2 |
|  | R | GCAGTAGGTGACAGCTTGGTTCG |  |  |  |
| PLCɡ2 | F | GTGTGAACCGAGGCATAGCAAGG | 54 | 84 bp | NC_0000746 |
|  | R | TAATGGAATGGAAGCGGCAGAAGC |  |  |  |
| FZD2 | F | ATGAAGCACGACGGCACCAAG | 54 | 111 bp | NM_020510.2 |
|  | R | GAAGTAGCAGGCGATGACGATGG |  |  |  |
| CAMK2A | F | GAAGTGAGGAAGAGGCAGGCAAG | 54 | 161 bp | NM_1774074 |
|  | R | GAGAGCAGATGAGAGCAGCAGTTC |  |  |  |
| GAPDH | F | TGGTGAAGGTCGGTGTGAAC | 57 | 231 bp | NM_001115114.1 |
|  | R | GCTCCTGGAAGATGGTGATGG |  |  |  |
| GHR | F | CCTACTGCATCAAGCTAACTAC | 56 | 132 bp | NM_001048178 |
|  | R | GAATCCCGGTCAAACTAATG |  |  |  |
| m-RAS | F | GAAGTGAGGAAGAGGCAGGCAAG | 61 | 178 bp | AF043581.1 |
|  | R | GAGAGCAGATGAGAGCAGCAGTTC |  |  |  |
| MAPK10 | F | GCGACATCCATCACCTGACTCTTC | 61 | 193 bp | XM_011249450.2 |
|  | R | GCCTCGGAATGCCAGCTTCTTC |  |  |  |
| ATF3 | F | ACAAGGCATGGCATGGCTTACAC | 61 | 131 bp | NM_007498.3 |
|  | R | TGTCTCCACAGCCACCAGATTCTC |  |  |  |
| EGR1 | F | GCTAGAGCTGCTGGCCTTGTTAG | 61 | 133 bp | NM_001964.3 |
|  | R | TCTCGGACTCCTGGTTCTGCTG |  |  |  |
